# The development of a method for the global health community to assess the proportion of food and beverage companies’ sales that are derived from unhealthy foods

**DOI:** 10.1186/s12992-023-00992-z

**Published:** 2023-12-01

**Authors:** Lauren Bandy, Jo Jewell, Madison Luick, Mike Rayner, Yuan Li, Katherine Shats, Susan Jebb, Suying Chang, Elizabeth Dunford

**Affiliations:** 1 Nuffield Department of Primary Care Health Sciences, University of Oxford, Oxford, UK; 2 UNICEF, 3 United Nations Plaza, New York, USA; 3 Nuffield Department of Population Health, University of Oxford, Oxford, UK; 4 The George Institute for Global Health, Beijing, China; 5 The George Institute for Global Health, UNSW, Sydney, Australia; 6 UNICEF Office for China, 12 Sanlitun Road, Chaoyang District, Beijing, China; 7 Department of Nutrition, Gillings School of Public Health, The University of North Carolina at Chapel Hill, Chapel Hill, NC USA

## Abstract

**Context:**

Corporate engagement with food and beverage companies who produce food associated with health harms is a divisive topic in the global nutrition community, with high-profile cases of conflict of interest increasingly coming under scrutiny. There is a need for an agreed method to support health organizations in deciding whether and how to engage with large food and beverage manufacturers.

**Aim:**

The aim of this study was to develop a method to quantify the proportion of sales from food and beverage companies that are derived from unhealthy foods to support organizations in determining which companies might be considered high-risk for engagement.

**Methods:**

The 2015 WHO Euro nutrient profile model was applied to 35,550 products from 1294 brands manufactured by the top 20 global food and beverage companies from seven countries (Australia, Brazil, China, India, South Africa, UK and USA). For the purpose of this study, products that met the WHO Euro criteria were classified as “healthier” and those that failed were classified as “unhealthy”. Products were grouped by brand and weighted by the brand’s value sales for 2020. The primary outcome was the proportion of each company’s sales that were classified as unhealthy and healthier by company and category.

**Results:**

Overall, 89% of the top 20 companies’ brand sales were classified as unhealthy. For every USD$10 spent on the top 20 companies’ brands, only $1.10 was spent on products considered healthier. All companies saw the majority of their sales come from unhealthy foods, including soft drinks, confectionery and snacks. None of Red Bull or Ferrero’s sales were classified as healthier and less than 5% of total sales were healthier for Mondelēz, Mars, and PepsiCo. Some companies had higher proportions of sales deriving from healthier products, including Grupo Bimbo (48%), Danone (34%) and Conagra (32%), although the majority of their sales were still derived from unhealthy foods.

**Discussion:**

The results presented in this study highlight the reliance the leading food and beverage companies have on sales of unhealthy products that are contributing to diet-related disease globally. The method and steps we have laid out here could be used by organizations in the global health community to identify companies that have conflicts of interest when it comes to engaging with governments, international organizations and public health bodies on issues of policy and regulation.

**Supplementary Information:**

The online version contains supplementary material available at 10.1186/s12992-023-00992-z.

## Background

Good nutrition is vital to health and development. There are only seven years remaining to achieve the United Nation’s Sustainable Development Goals of ending food insecurity and malnutrition in all forms [1], yet in recent years progress has slowed and global food security has deteriorated [2]; 200 million children under the age of five are still affected by stunting or wasting, and an estimated 39 million are overweight – this number grows to 340 million overweight children aged 5–19 years [3]. Two in five adults are overweight or obese globally [3] and nearly three quarters of overweight children live in low- and middle-income countries [4].

Multinational food producers, manufacturers and retailers dominate the global food system. While new agricultural techniques and food processing technologies have increased affordability and accessibility to foods rich in essential nutrients [5], it also means that diets across the globe have become dominated by highly processed unhealthy foods [6–8]. Many multinational food companies have committed to socially responsible initiatives, such as not marketing products high in fat, sugar and salt (HFSS) to children, providing labelling on the front and back of the pack and moderating nutrition claims [9, 10], although these voluntary agreements are not considered to be effective at safeguarding the population’s health [11]. Initiatives such as the Access to Nutrition Index have attempted to independently assess the world’s largest global food and beverage manufacturers with regards to governance and management; the production and distribution of healthy, affordable, accessible products; and how they influence consumer choices and behavior [12].

The public health community – be that UN-agencies, research institutions, scientific committees or non-governmental organizations – already treats the food and beverage industry with caution, not only to protect their work from conflicts of interest, but also to avoid reputational risk. Recently there have been several cases of food and beverage companies being involved with high-profile events and organizations that have led to negative global media attention with consequent risks to their core missions, including through the UN Food Systems Summit [13], COP27 [14], the International Union of Nutrition Scientists [15], the Academy of Nutrition and Dietetics [16] and the European Congress on Obesity [17].

Previous research has established methods to assess the healthiness of the leading food and beverage companies’ portfolios [18] [19] [20] but a substantiated method for the global health community to identify the food and beverage companies whose core business – their product portfolios and sales – contribute the most towards unhealthy diets and diet-related disease globally is needed. This is especially true of large multi-national companies; the retail sales of the top 20 global packaged food and soft drinks companies exceeded US$7.7 billion in 2022, representing 22% of global market share [21], and it is these big companies and their brands that are most likely to approach international organizations with opportunities of fundraising, sponsorship, partnership and policy engagement. There is a need from the international nutrition community for a quantitative method that can be used by organizations to guide their principles of engagement with the food industry.

Nutrient profiling is “the science of classifying and ranking foods according to their nutritional composition for reasons related to preventing disease and promoting health” [22]. The WHO Euro nutrient profile model (NPM) is designed to be used by governments to identify which foods may and may not be marketed to children [22]. The model considers whether products exceed thresholds for energy, total fat, saturated fat, trans fats, salt, and total sugars and whether the products contain added sugars or non-sugar sweeteners (NSS). If products exceed a threshold or contain added sugars or NSS, they cannot be marketed to children. The WHO Euro NPM is often seen as the “standard” NPM [22] and has been widely used in the literature, including in validation studies [23].

The objective of this study was to develop a method that quantifies what proportion of a food and beverage company’s core business comes from unhealthy products using the WHO Euro NPM, with the aim of informing global health organizations’ principles of engagement.

## Methods

### Data sources

Data on the value sales of packaged food and soft drink brands, expressed as United States Dollars (USD), for the top 20 global companies were sourced and used under licence from Passport GMID, Euromonitor International and accessed via the Bodleian Library, University of Oxford [24]. For brevity, this is hereafter referred to as ‘sales data’. The sales data are subject to licencing terms and therefore cannot be made open access with this paper.

The FoodSwitch database from The George Institute for Global Health was used to source nutrition composition data. FoodSwitch captures images of packaged foods and beverages using a bespoke mobile application, allowing for the extraction and collation of key food labelling and composition data [25]. Product information is controlled with an established quality assurance protocol, reviewed and categorised into the database and has been explained in further detail previously [25]. FoodSwitch data were used to source data on nutrient content per 100 g/ml (including energy (kcal/kJ), carbohydrate, total sugars, protein, fat, saturated fat, fibre and salt) and ingredients information.

### Data analysis

Sales data were used to identify the top 20 packaged food and soft drink companies globally. The brands manufactured by each of these companies were identified in the FoodSwitch database for seven countries: Australia, Brazil, China, India, South Africa, UK and USA. These seven countries were selected as they are a leading market in each geographic region of the world.

The WHO Euro NPM is used by governments in the European region to test whether or not foods should be subject to marketing restrictions to children because they are unhealthy. The First Edition (2015) of the WHO Euro NPM covers 20 pre-packaged food categories that are designed for consumption by children aged 5 years and over [22]. Prepared baby foods and infant formulas are not covered by the model and were excluded from this analysis, given they are not for the consumption of the whole population and are subject to their own marketing restrictions. Sales data on whole fresh and frozen meat was not available and also excluded. Non-food brands that are manufactured by the top 20 companies, such as pet food and home care products, and alcohol and low-alcohol products, were also excluded from this study.

All foods in the FoodSwitch database had a WHO Euro category pre-assigned and each brand in the sales database was also assigned a WHO Euro category name. The WHO Euro NPM thresholds for energy, total fat, saturated fat, total sugars, added sugars, NSS and salt were applied to each product on a per 100 g basis to assess if it would pass the model and be eligible to market to children – these thresholds can be found in Additional file 1. For the purpose of this study, if a product failed the model, it was classified as ‘unhealthy’. If a product passed the model, it was classified as ‘healthier’.

The main outcome variable was the proportion of each company’s value sales (USD) that was derived from brands that were classified as ‘unhealthy’ by the NPM. Analyses for this study were completed in RStudio version 2022.07.2.

### Calculating the proportion of unhealthy sales by company

We weighted each product by its brand sales values. Where brands were matched with more than one product variant (e.g. different flavor variants), each product was considered to have equal sales, that is the brand sales value was divided by the total number of products in that brand.

**Fig. 1:**
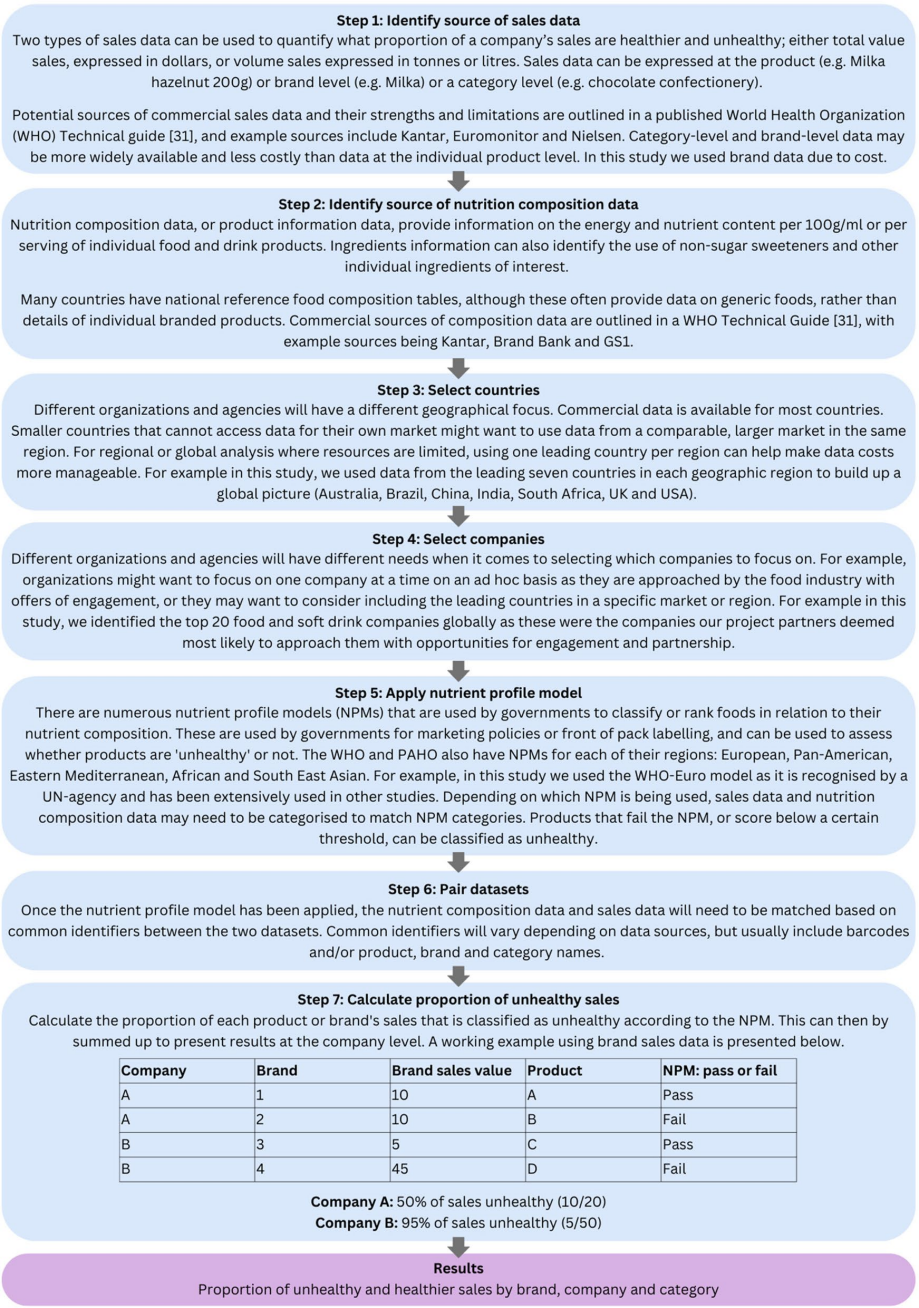
Detailed guide to developing a tool to assess proportion of companies’ sales from healthier and unhealthy foods

To aid other organizations in being able to use and adapt these methods for their own specific needs, we present a step-by-step guide—including alternative sources of data and a working example of how to calculate the proportion of unhealthy sales—in Fig. 1 below.

## Results

A total of 35,550 products belonging to 1294 brands manufactured by the top 20 companies were included in the analysis for all countries (Table 1). A total of 316 brands, representing 12.1% of value sales, could not be matched to nutrition information and were excluded from this analysis.

**Table 1:** The number of products and brands included in analysis by company

**Company name**	**Number of brands**	**Number of products**	**Number of countries**	**Categories included**
Campbell Soup	37	2633	3	9
Coca-Cola	114	1749	7	6
Conagra	46	2434	3	8
Danone	38	1197	5	5
Ferrero	59	2229	7	2
General Mills	53	2232	7	11
Grupo Bimbo	14	363	4	3
Kellogg	66	1128	7	5
Keurig Dr Pepper	14	661	2	2
Kraft Heinz	85	3373	7	11
Lactalis	53	923	7	8
Mars	102	2955	7	9
Mengniu	16	325	1	5
Mondelēz	150	2850	7	8
Nestlé	157	2908	7	11
PepsiCo	160	4098	7	10
Red Bull	10	91	7	1
Suntory	17	180	4	5
Unilever	91	3007	7	9
Yili	12	214	1	3
**Total**	**1294**	**35,550**	**7**	**19**

**Fig. 2:**
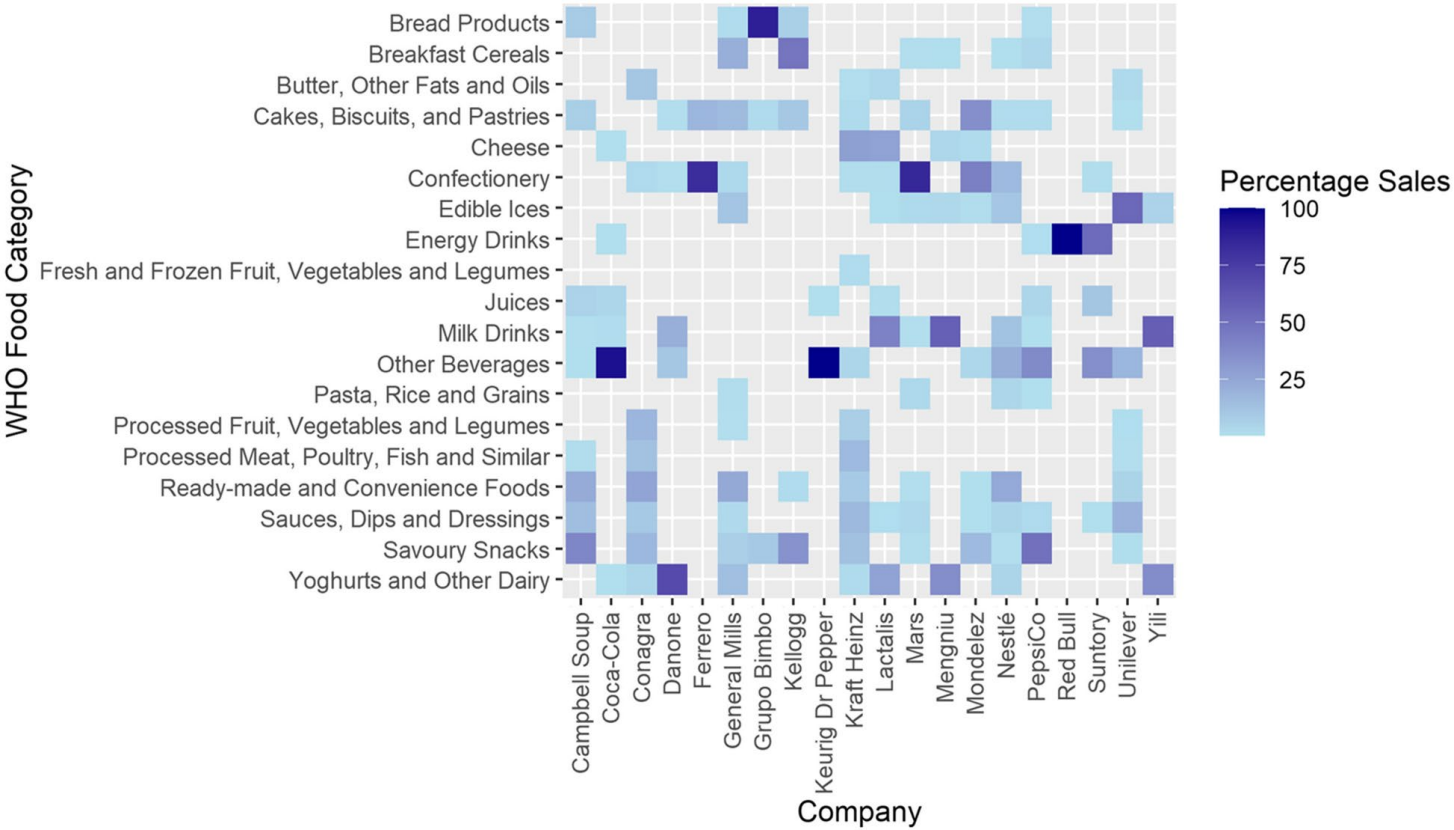
Proportion of revenue by product category and company

There was great heterogeneity in the level of diversity of companies’ product portfolios (Fig. 2). Revenue for some companies e.g. like Red Bull and Ferrero, came from only one or two categories, while others had a much more diverse product range e.g. Nestlé and Kraft Heinz.

Overall, 89%% of the top 20 global food and beverage companies’ brand sales were classified as unhealthy according to the WHO Euro NPM. This means that for every USD$10 spent on the top 20 companies’ brands, only $1.10 was spent on products that are healthy enough to be marketed to children under the WHO Euro NPM.

**Fig. 3:**
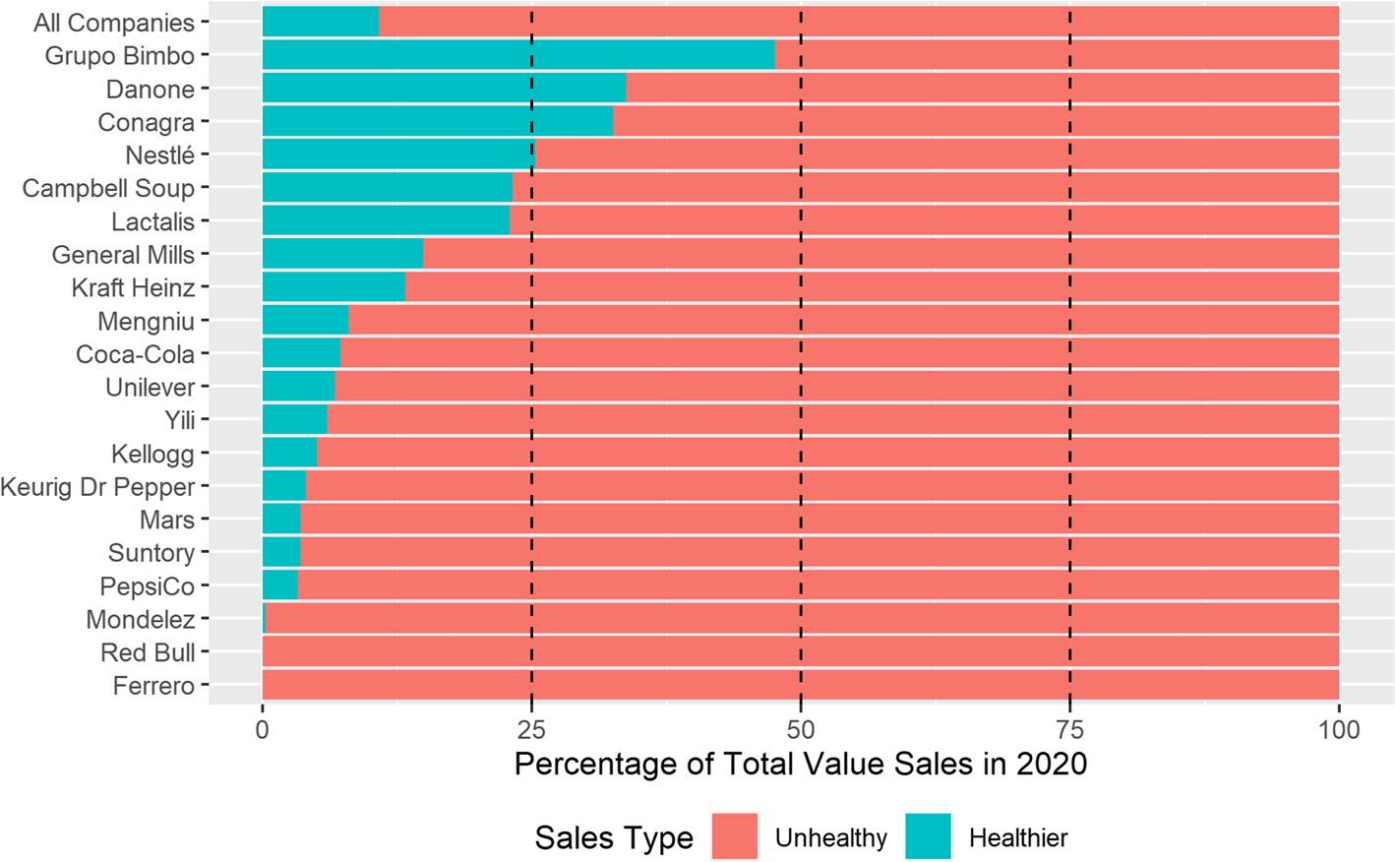
Proportion of brand sales that are classified as healthier and unhealthy by company

All of Red Bull or Ferrero’s brands were classified as unhealthy, while 95% of sales came from unhealthy products for five companies – Mondelēz, PepsiCo, Suntory, Mars, and Keurig Dr Pepper whose portfolios and sales are dominated by confectionery, biscuits and cakes, and soft drinks. Grupo Bimbo was the company with the highest proportion of sales deriving from healthier products, at 48%, followed by Danone (34%) and Conagra (33%) (Fig. 3).

**Fig. 4:**
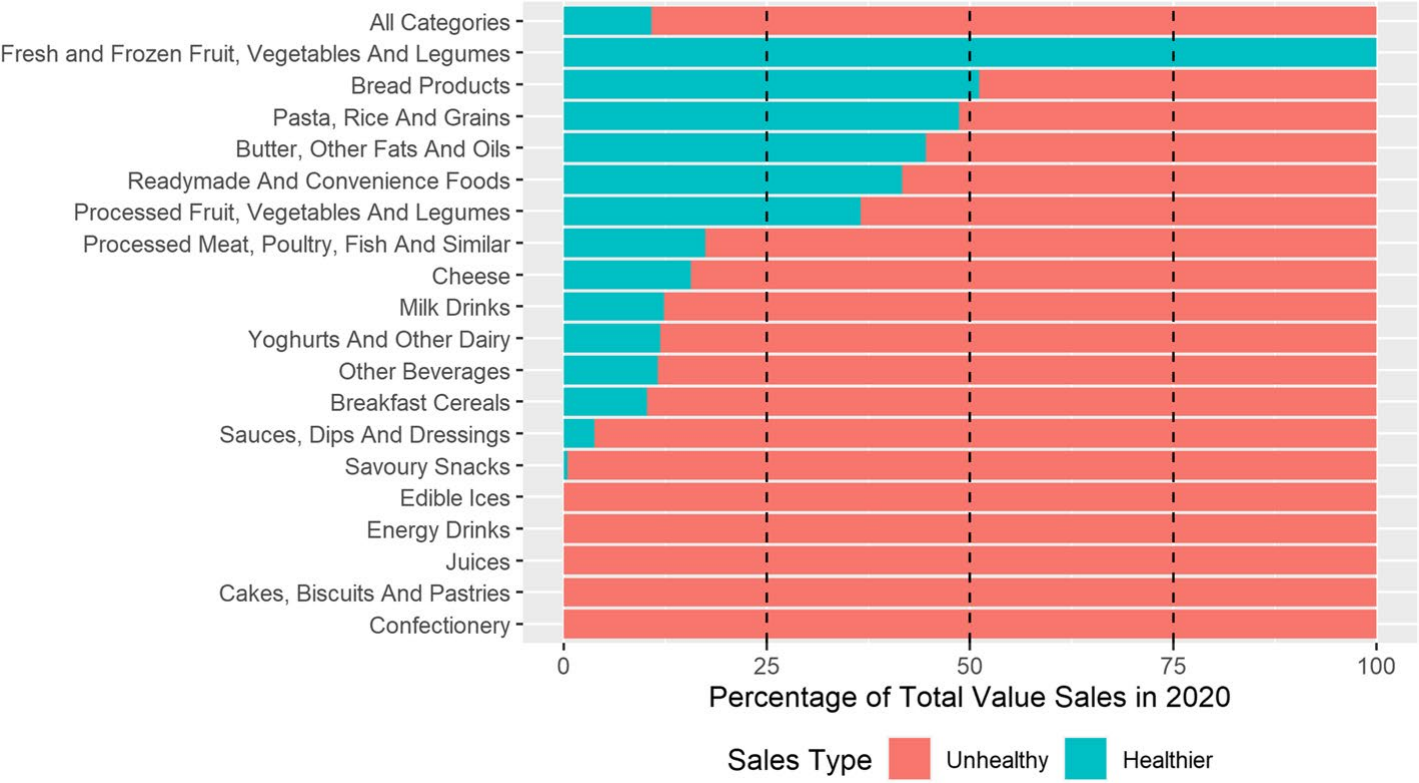
Proportion of sales classified as healthier and unhealthy by category

The WHO-Euro NPM does not permit the marketing of chocolate or sugar confectionery; cakes, biscuits and other sweet bakery products; edible ices and ice cream; energy drinks; and juices, therefore all sales from these categories were classified as unhealthy (Fig. 4). Category level results are presented for each company in Additional file 2. There were two product categories where the majority of sales were for healthier products, fresh and frozen fruit and vegetables (100%) and bread products (51%). Pasta, rice and grains (49%), butter, fats and oils (46%) and readymade and convenience foods (42%) also had a relatively higher proportion of healthier sales. However, these categories represent a small amount of total revenue when compared to the leading categories: other beverages, savoury snacks and confectionery. Absolute sales by category and company are presented in Additional file 3.

## Discussion

The product portfolios of the world’s top 20 food and beverage companies are dominated by confectionery, savory snacks, biscuits, cakes and soft drinks, and 89% of overall sales were from unhealthy foods that are high in sugar, fat and salt. For every $10 spent on these companies’ products in 2020, only $1.10 was spent on healthier products. Companies dependent on soft drinks and confectionery saw very little – or in the case of Red Bull and Ferrero, none – of their sales derive from brands classed as healthier. Healthier categories, such as fresh fruit and vegetable products and breads and other staples, represented a small proportion of overall sales.

It is increasingly important to protect the fundamental goals and reputations of public health bodies and other organizations from association with food and beverage companies whose revenue derives from products that contribute directly to the ill-health of populations worldwide. The methods presented in this paper provide an objective and transparent means to assess companies’ product portfolios and sales. We propose that there are two main ways this paper can be used by other organizations. The first is that the method could be adapted for use in other countries, with different sales data, composition databases and nutrient profile models that are specific to the organization’s needs and country context. To aid this process, we have presented a detailed step-by-step guide to the method (Fig. 1). The second is that organizations could use the global results presented here in this paper directly – they could be used to inform their engagement policies and identify conflicts of interest. These methods could also be used to inform advocacy groups and policymakers about the extent to which companies are contributing towards poor diets, and better hold them to account. The finance community could also use these methods when considering risk of investment and how vulnerable food and beverage companies are to stricter health-related policies – including fiscal measures – in the future.

However, it should be noted that the method described here has only covered one element of a company’s business practices: the healthiness of its product portfolio and sales. Other elements that may be of importance to other organizations and agencies include the marketing practices, lobbying practices and influence on health and diet policy at an international, national and regional level of each individual company. Existing resources such as ATNI’s *Global Index* and *Country Spotlight Reports* [12] provide some insights into these practices. Organizations in the global health and nutrition community could also consider setting a threshold for the proportion of unhealthy sales food and beverage companies must meet in order to engage with them. For example, it could be an organisation says the minority of a company’s sales must be from unhealthy foods (e.g. 49%, 25% or < 10%) or if repeated over time, could be based on an improvement (e.g. a reduction of unhealthy sales by 50% over a given time period). Setting and validating these thresholds is something that warrants further research, and could encourage companies to improve the healthiness of their sales.

### Strengths and weaknesses

While a theoretical typology for public–private engagement in the nutrition sector has been published [26] and UNICEF has published guidance on engaging with the food and beverage industry [27], this is the first time an objective and quantitative tool to inform engagement decisions has been published. This study has wide geographical reach and includes data from seven food and beverage markets from each region of the globe. Including additional countries in the future would give further insight into how leading multinational companies’ sales vary geographically. Repeating this study annually would allow for companies’ progress to be tracked over time. While it is the largest multinational companies that are most likely to seek public–private partnerships and memberships in multi-stakeholder platforms, this study is limited by only including 20 companies. Nationally, a different set of local manufacturers may approach organizations with offers of corporate engagement which are not covered here. We were not able to identify any examples of companies with the majority of their sales coming from healthier products that might be considered lower risk for organizations to engage with. Previous work in nutrient profiling has demonstrated that companies with portfolios dominated by dairy products often have overall “healthier” product offerings [28] and it may be that in countries where dairy companies dominate (such as in Europe), there may be cases of best practice to identify. However, there may be other factors that influence engagement principles with dairy companies, including their compliance with the international code of marketing of breast milk substitutes.

Euromonitor sales data cover the brands sold from packaged food and soft drink categories in 80 countries globally. The main limitation is that brand level sales data (e.g. Yoplait yoghurts) had to be paired with the nutrition information for individual products (e.g. Yoplait original and Yoplait light). This meant that where a brand has multiple product flavors/variants, sales of each individual product was weighted equally, when it may be that one product variant/flavor represents more sales. Ideally, product level, rather than brand-level, sales data could be used, although there are limited data sources for product level sales, and those that do exist have their own limitations, including being expensive, having publication limitations and high levels of imputation [29, 30]. This could be overcome if companies were more transparent with their data, although they are unlikely to want to report individual product sales for fear of disclosing information to their competitors. Reporting percentages instead of absolute values ($) could help overcome this.

There were 316 brands that were excluded from this analysis because they could not be paired with nutrition composition data. This represents 20.0% of the total number of brands, but only 12.1% of total sales, suggesting that these were smaller brands with smaller sales. There was geographical variation, with 97% of sales data matched with nutrition composition data in Brazil, compared to just 62% in China. These differences are down to three reasons: errors in the sales data, where brands that are no longer sold in that market are still listed; brands not being captured in the FoodSwitch database; or differences in the translation of product names between the sales and composition databases. In the future, missing data could be reduced by using product-level sales data that includes barcode, so that products between databases could be better matched, although more granular sources of sales data are often prohibitively expensive [29, 30].

There are several NPMs that could have been used in this study. The WHO has a different model for each of its regions [31–33], and the Pan-American Health organization (PAHO) also has an NPM [33]. There are also other widely used models, including the UK Ofcom model [34], Nutri-Score which is used widely across Europe [35], and Australia and New Zealand’s Health Star Rating system [36]. The WHO Euro NPM was used because it is approved by a UN-agency, is regularly used by researchers and using a single model allowed for a unified and comparable method across countries globally. However, depending on the geographies included, a country specific scheme may be preferable. It is important to note that some other models, such as the PAHO NPM, are stricter than the WHO Euro NPM [37] while others e.g. UK NPM, that do not have thresholds for NSS and therefore class zero-calories soft drinks as healthier, are less so.

### Comparison to other studies

There are other initiatives that score and rank food and beverage companies based on their performance in nutrition and health which have reached broadly similar conclusions that the world’s largest food and beverage companies are focused on the sale of less healthy products. The Access to Nutrition Initiative (ATNI) [28] aims to rank global food and beverage companies on their contribution to addressing malnutrition, including overweight and obesity, undernutrition and micronutrient deficiencies based on a range of areas, including commitments and practices in terms of governance and management, healthiness of their products based on the Health Star Rating, and how they influence consumer behavior through labelling and marketing practices. ATNI’s most recent Global Product Profile examined the healthiness of the 25 largest companies globally in 25 countries and found that overall 31% of products would be considered “healthier” using the Australasian Health Star Rating system, and that by using the locally-appropriate WHO NPM that only 9% of products would be considered healthier. These results seem in line with the findings presented here and highlight that using a different NPM is unlikely to change the main observation the majority of the world’s top food and beverage companies’ sales are from unhealthy products.

INFORMAS (International Network on Food and Obesity/NCD Research, Monitoring and Action) [38] has produced a series of company scorecards that assess companies’ impact on the food system using the Business Impact Assessment on obesity and nutrition (BIA-Obesity) tool, with a focus on companies’ self-reported nutrition policies, commitments, disclosure and performance [39]. While these metrics are based on business practices and companies’ commitment to nutrition-related policies, they are not directly comparable to the quantitative analysis presented in this study.

## Conclusion

This study provides an objective and transparent method to evaluate the nutritional risk profile of food and beverage companies. It shows that the world’s largest businesses in the sector are heavily dependent on revenue that is derived from the sales of unhealthy products. This information may be valuable to organisations wishing to assess the risk when considering partnerships. It can also be used by public health organisations for advocacy activities or to monitor and report on company commitments to move towards healthier business practices.

### Supplementary Information

**Additional file 1. **WHO European Nutrient Profile Model categories and nutrient thresholds.

**Additional file 2. **Proportion (%) of each company’s sales that are classified as unhealthy by category.

**Additional file 3: Supplementary Figure 1. **Absolute value sales (US$ million) that are unhealthy and healthier by company. **Supplementary Figure 2. **Absolute value sales (US$ million) that are unhealthy and healthier by category.

## Data Availability

The data that support the findings of this study are available from Euromonitor International and The George Institute for Global Health, but restrictions apply to the availability of these data, which were used under license for the current study, and so are not publicly available. Data are however available from the corresponding author upon reasonable request and with permission of Euromonitor International and The George Institute for Global Health.

## Abbreviations

HFSS: High fat, sugar and/or salt
NCD: Non-communicable disease
NPM: Nutrient profile model
UNICEF: United Nations Children’s Fund
WHO: World Health Organization
