# Supplementary material for: The development of a method for the global health community to assess the proportion of food and beverage companies’ sales that are derived from unhealthy foods
Source: Global Health. 2023 Dec 1;19:94. doi: 10.1186/s12992-023-00992-z (PMC10690999; doi:10.1186/s12992-023-00992-z)
Supplement: Supplementary file 1 — Additional file 1. WHO European Nutrient Profile Model categories and nutrient thresholds. [file 12992_2023_992_MOESM1_ESM.docx]

## Additional File 1: WHO European Nutrient Profile Model categories and nutrient thresholds

|  | **Marketing not permitted if product exceeds, 100g** | | | | | | |
| --- | --- | --- | --- | --- | --- | --- | --- |
| **Food Category** | **Total fat (g)** | **Saturated fat (g)** | **Total sugars (g)** | **Added sugars (g)** | **Non-sugar sweeteners (NSS) (g)** | **Salt (g)** | **Energy (kcal)** |
| 1. Chocolate and sugar confectionery, energy bars and sweet toppings and desserts | Not permitted | | | | | | |
| 2. Cakes, sweet biscuits and pastries; other sweet bakery wares, and dry mixes for making such | Not permitted | | | | | | |
| 3. Savoury snacks |  |  |  | 0 |  | 0.1 |  |
| 4. Beverages |  |  |  |  |  |  |  |
| a) Juices | Not permitted | | | | | | |
| b) Milk drinks | 2.5 |  |  | 0 | 0 |  |  |
| c) Energy drinks | Not permitted | | | | | | |
| d) Other beverages |  |  |  | 0 | 0 |  |  |
| 5. Edible ices | Not permitted | | | | | | |
| 6. Breakfast cereals | 10 |  | 15 |  |  | 1.6 |  |
| 7. Yoghurts, sour milk, cream and other similar foods | 2.5 | 2 | 10 |  |  | 1.2 |  |
| 8. Cheese | 20 |  |  |  |  | 1.3 |  |
| 9. Ready-made and convenience foods and composite dishes | 10 | 4 | 10 |  |  | 1.2 | 225 |
| 10. Butter and other fats and oils |  | 20 |  |  |  | 1.3 |  |
| 11. Bread, bread products and crisp breads | 10 |  | 10 |  |  | 1.2 |  |
| 12. Fresh or dried pasta, rice and grains | 10 |  | 10 |  |  | 1.2 |  |
| 13. Fresh and frozen meat, poultry fish and similar | Permitted | | | | | | |
| 14. Processed meat, poultry, fish and similar | 20 |  |  |  |  | 1.7 |  |
| 15. Fresh and frozen fruit, vegetables and legumes | Permitted | | | | | | |
| 16. Processed fruit, vegetables and legumes | 5 |  | 10 | 0 |  | 1 |  |
| 17. Sauces, dips and dressings | 10 |  | 0 |  |  | 1 |  |

Adapted from WHO Regional Office for Europe Nutrient Profile Model (First Edition, 2015) [22]
