# Supplementary material for: The development of a method for the global health community to assess the proportion of food and beverage companies’ sales that are derived from unhealthy foods
Source: Global Health. 2023 Dec 1;19:94. doi: 10.1186/s12992-023-00992-z (PMC10690999; doi:10.1186/s12992-023-00992-z)

## Additional File 2: Proportion (%) of each company’s sales that are classified as unhealthy by category


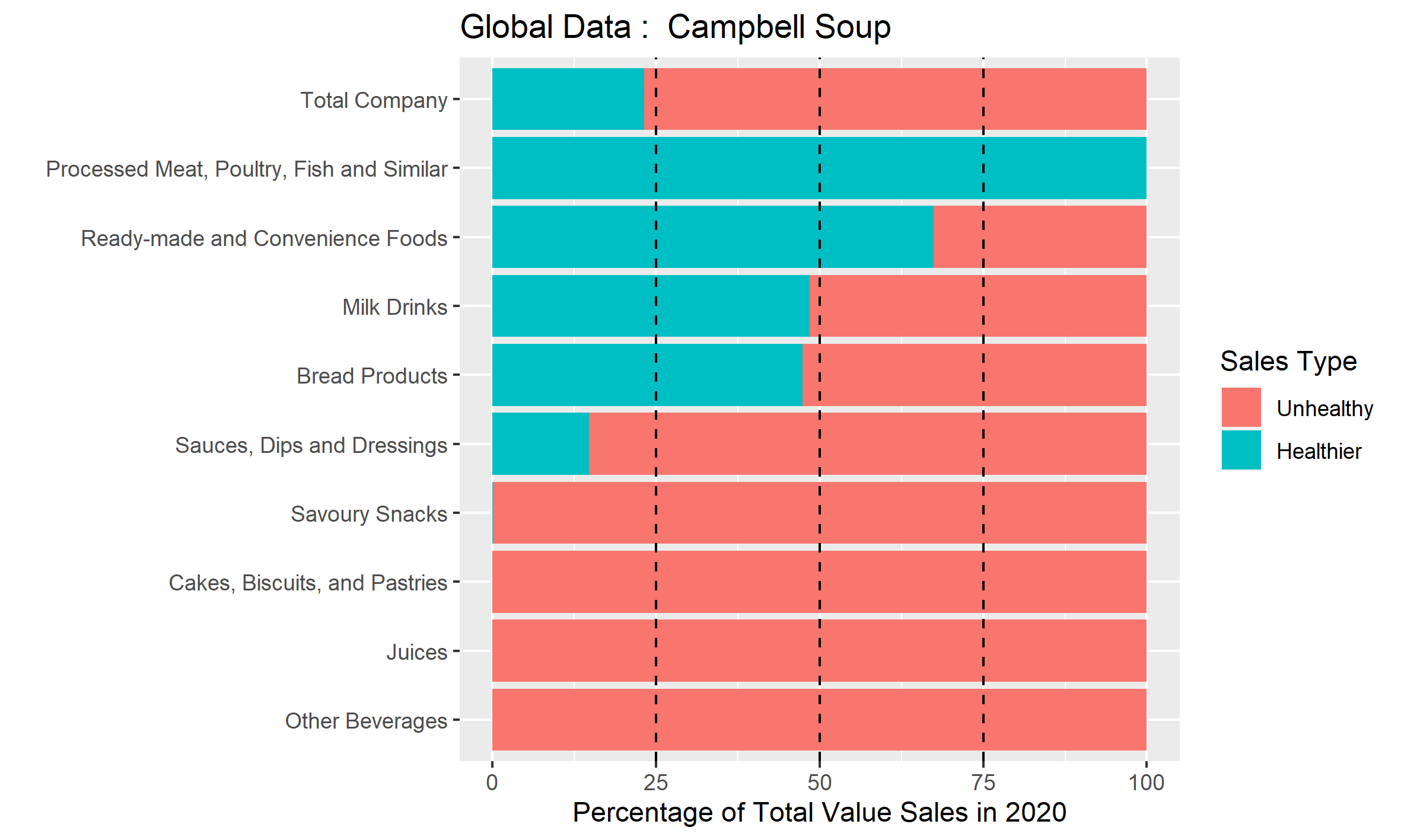


**
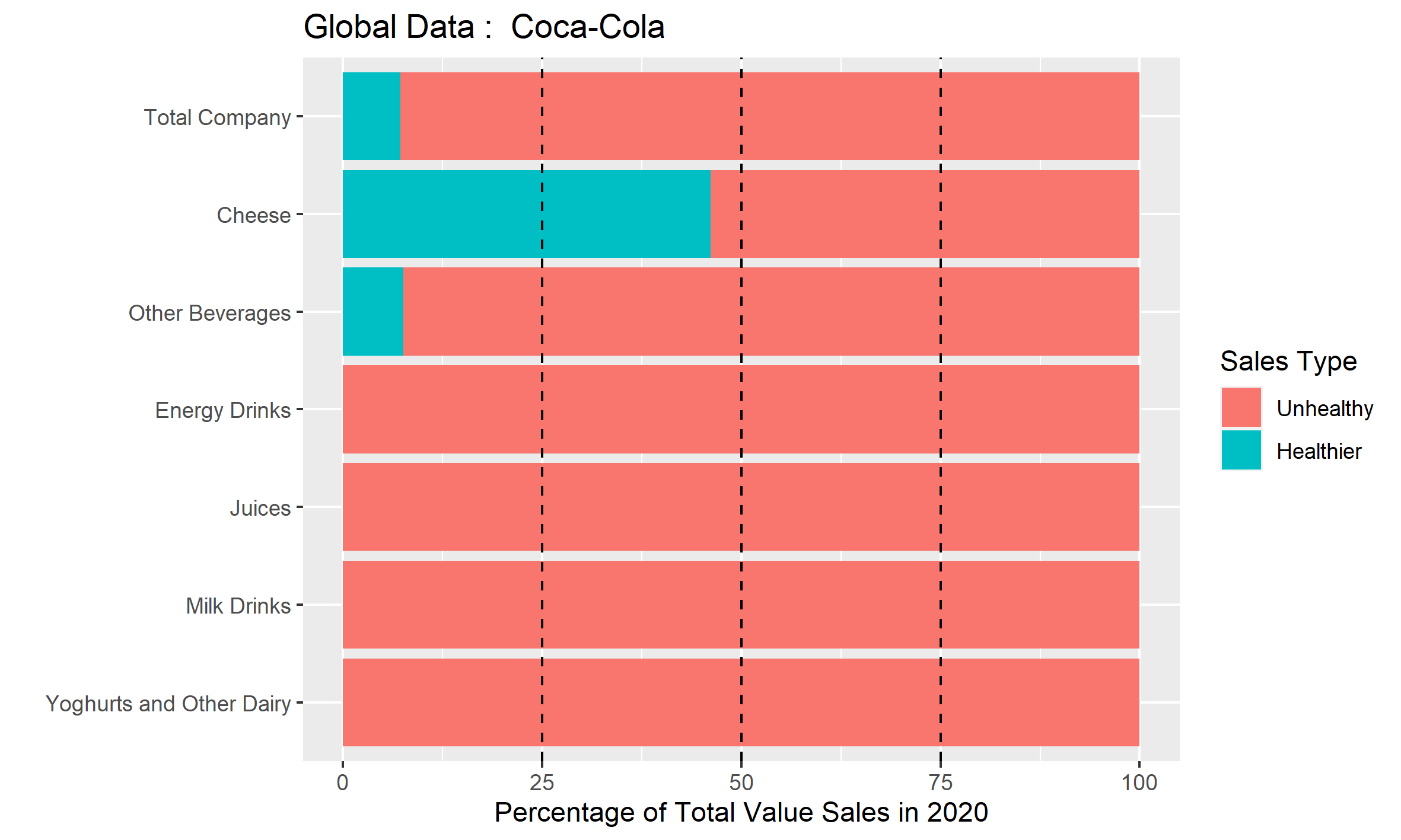
**

**
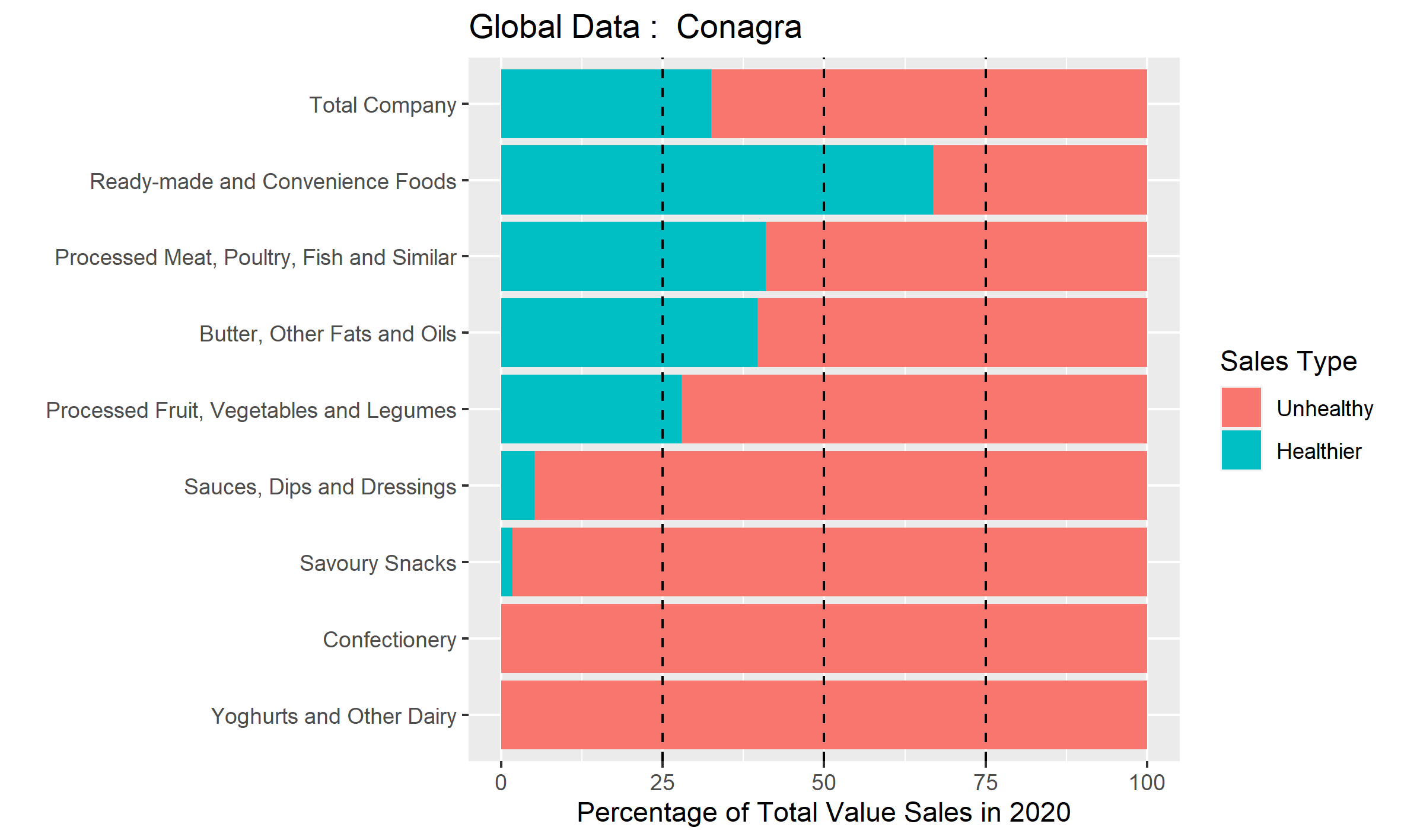
**

**
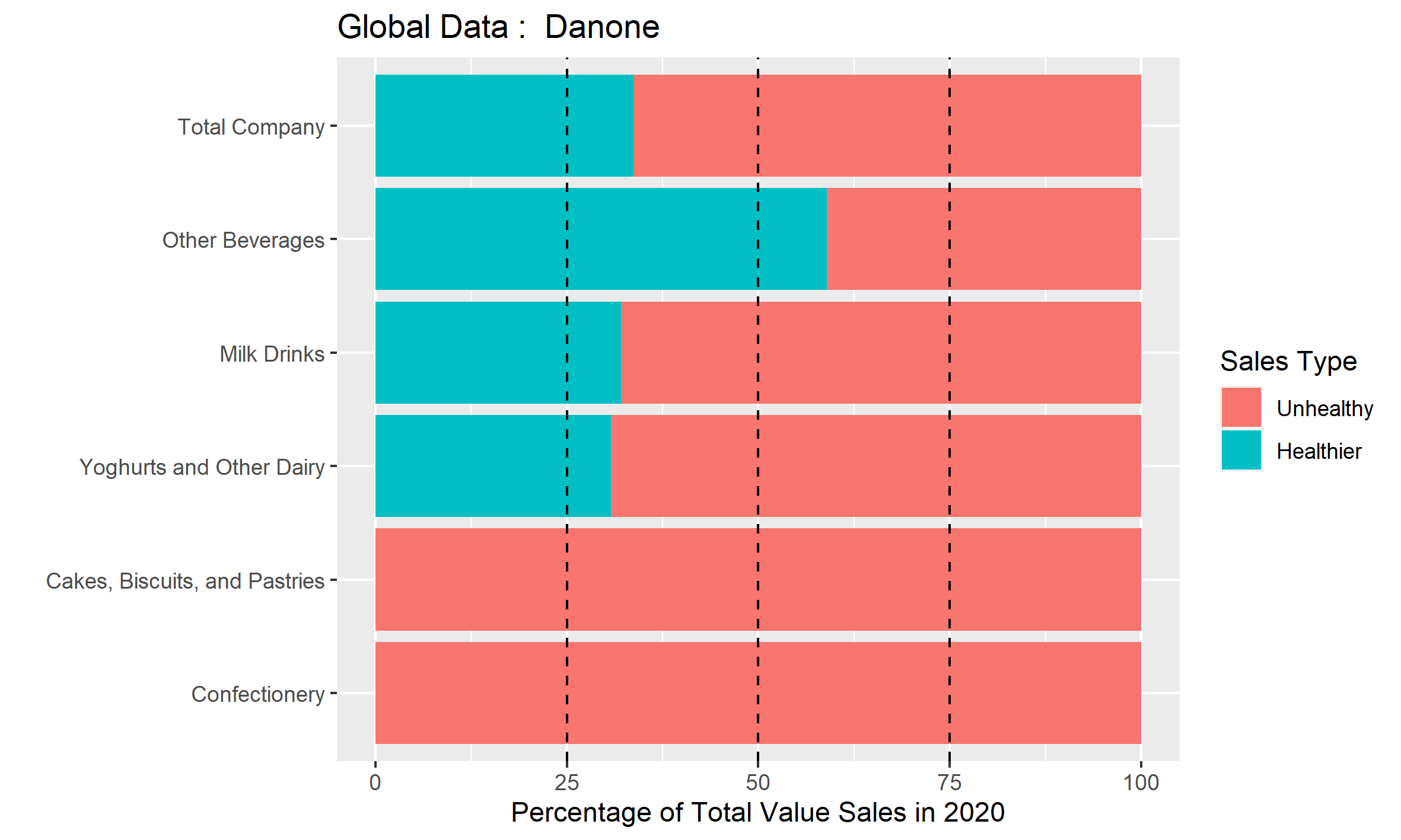
**

**
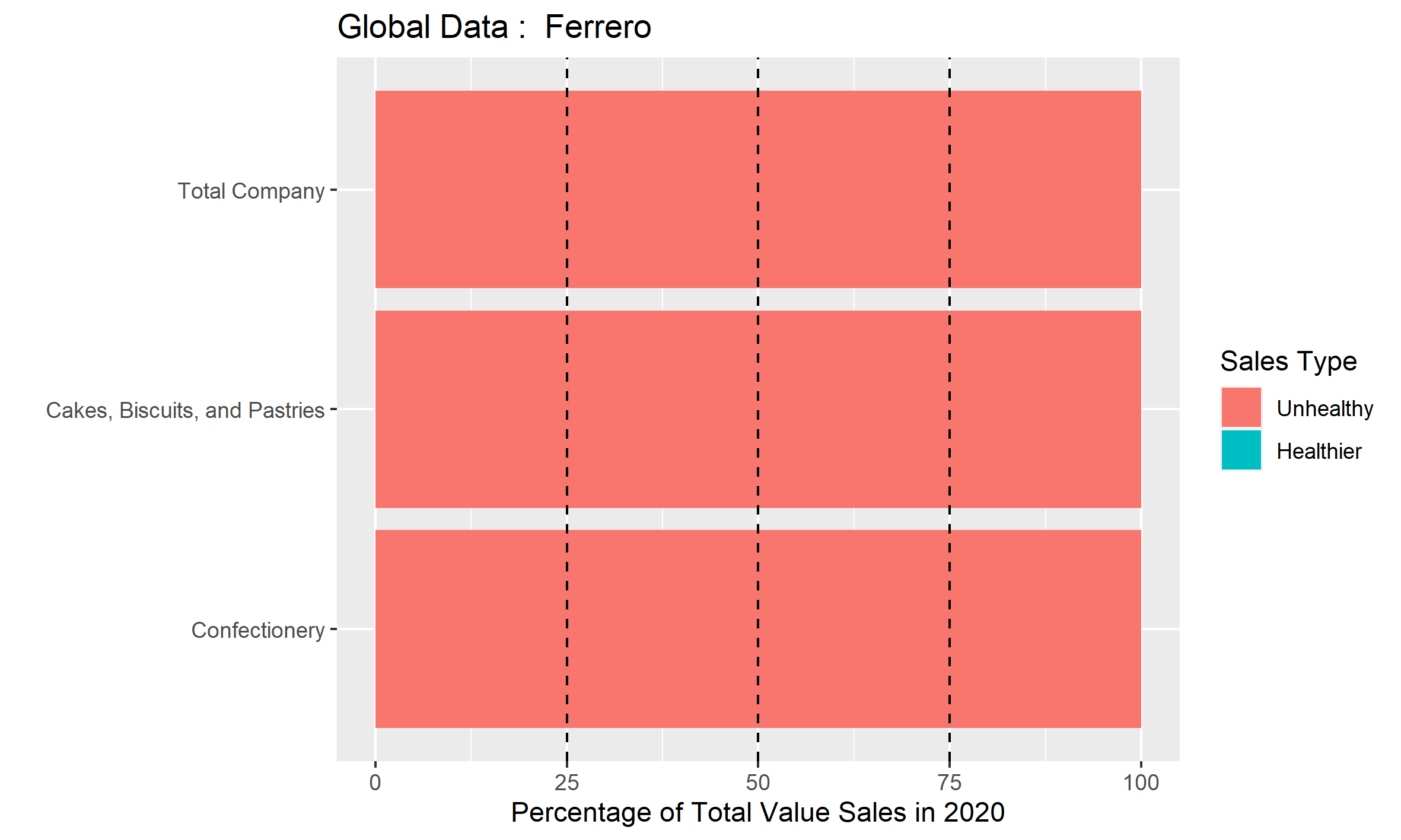
**

**
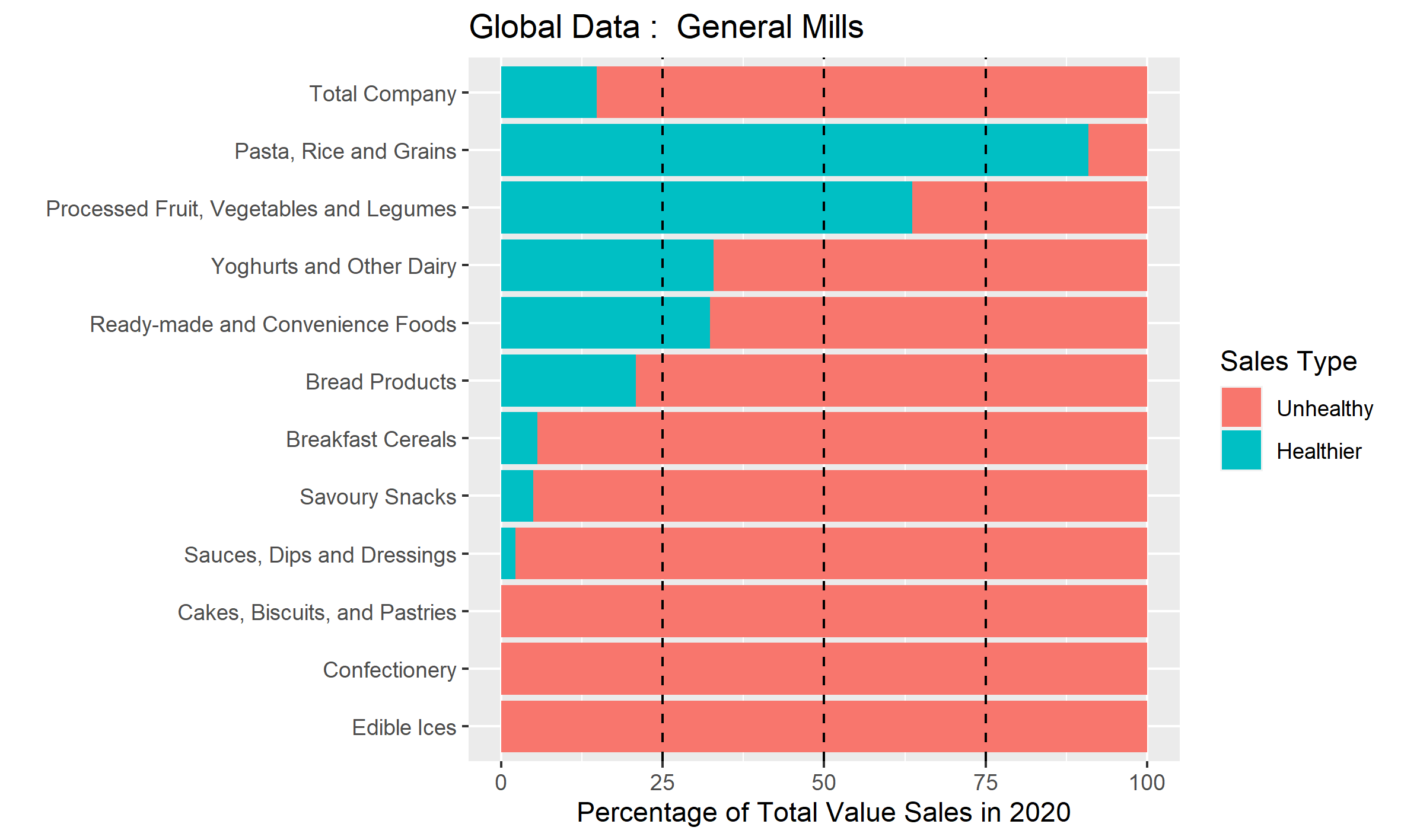
**

**
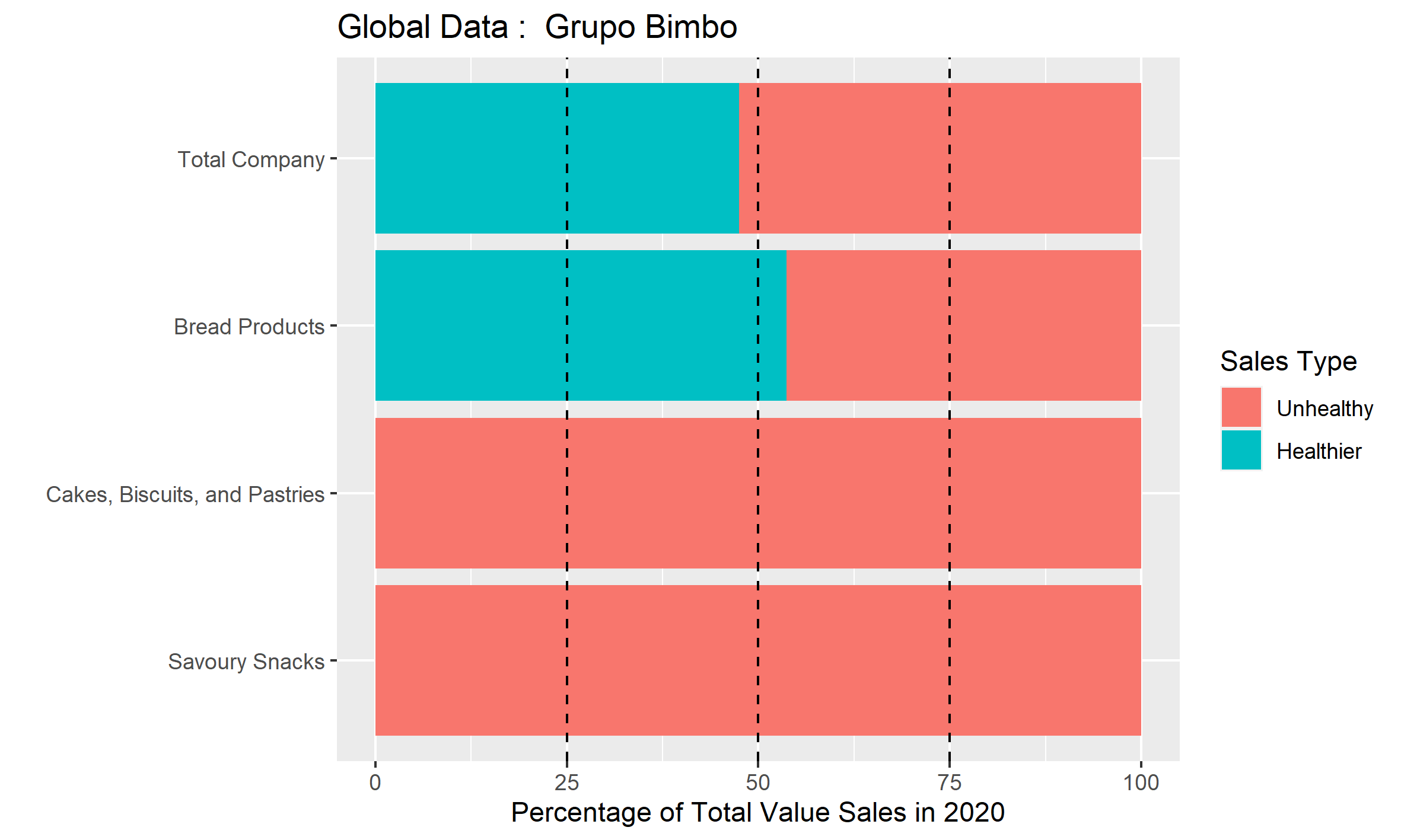
**

**
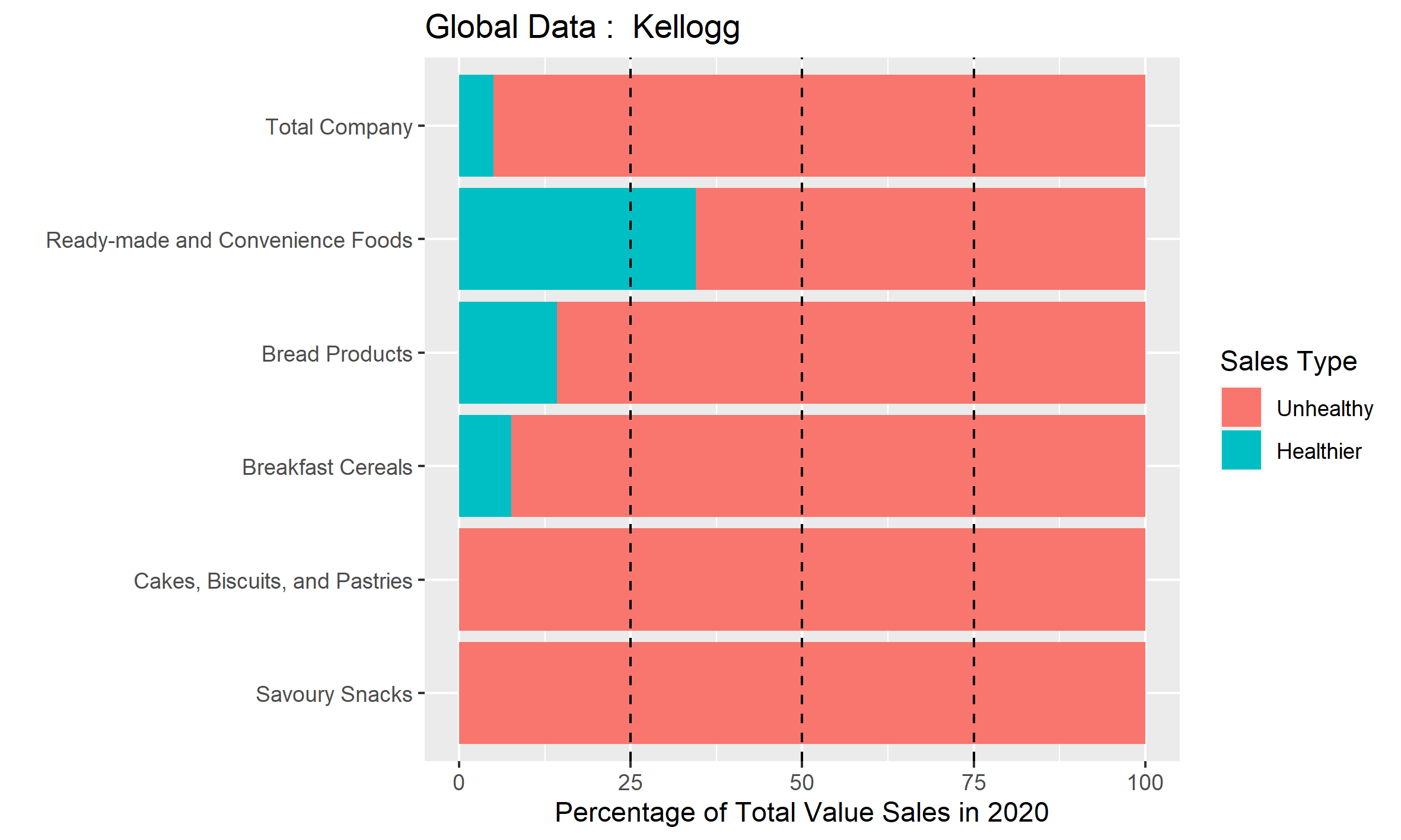
**

**
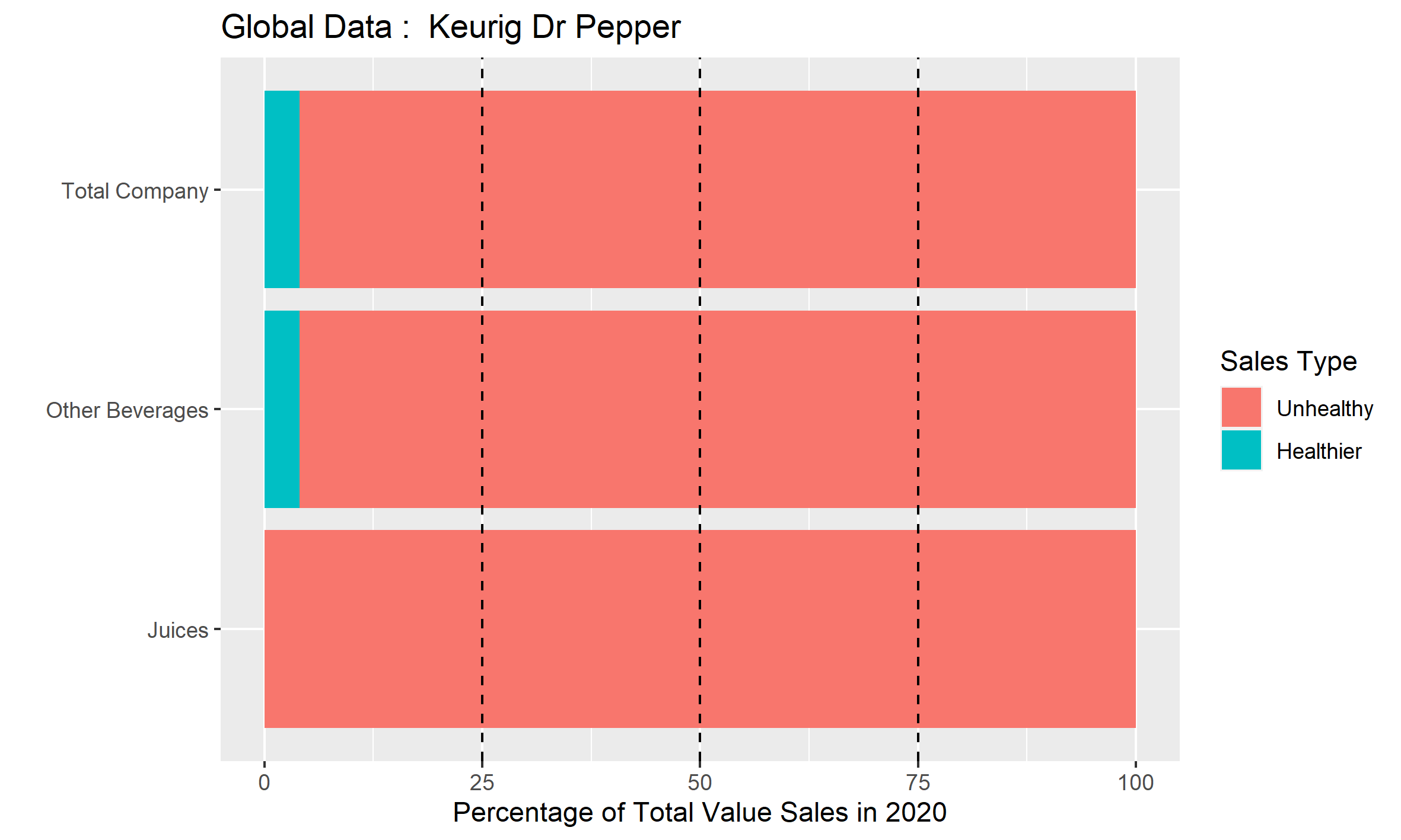
**

**
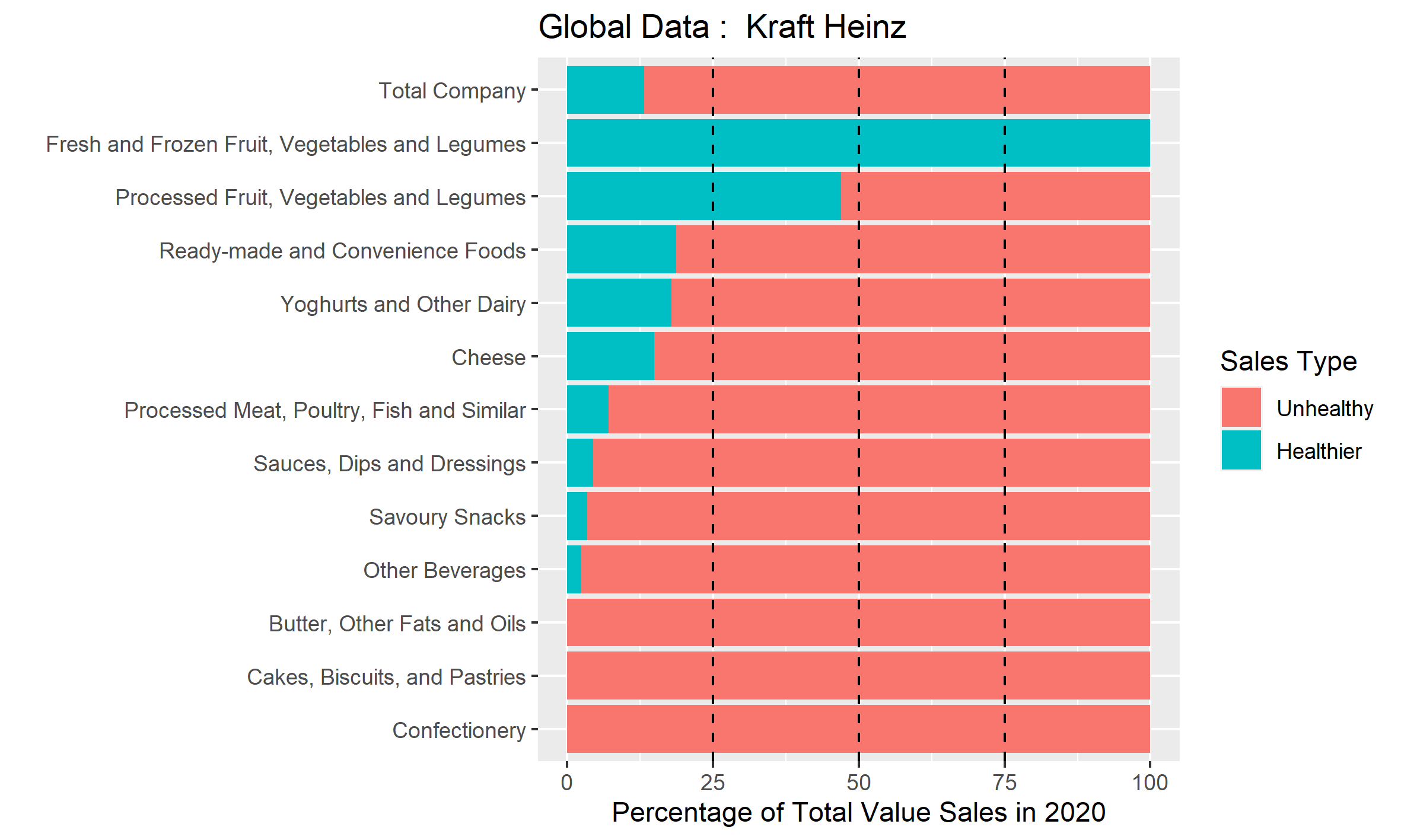
**

**
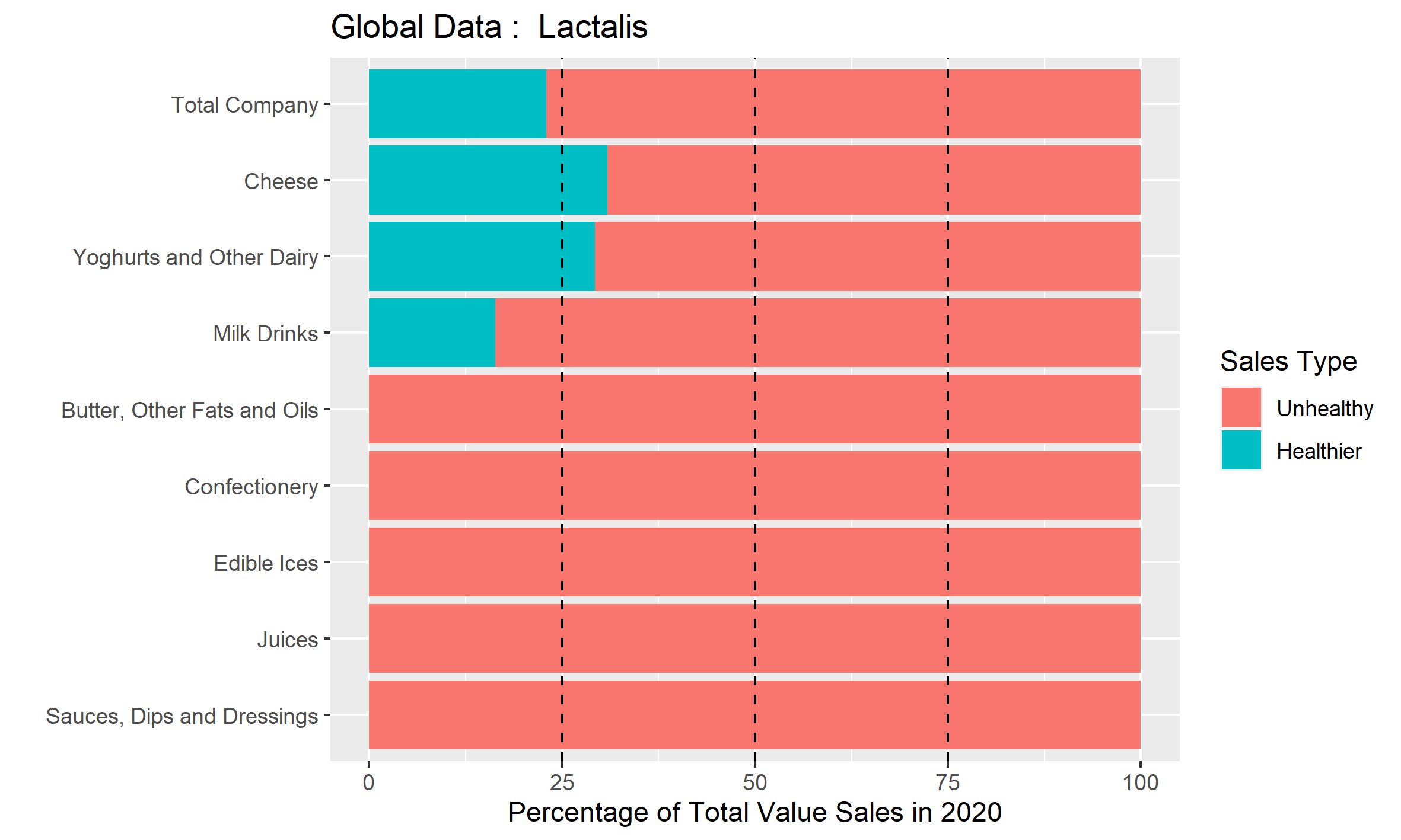
**

**
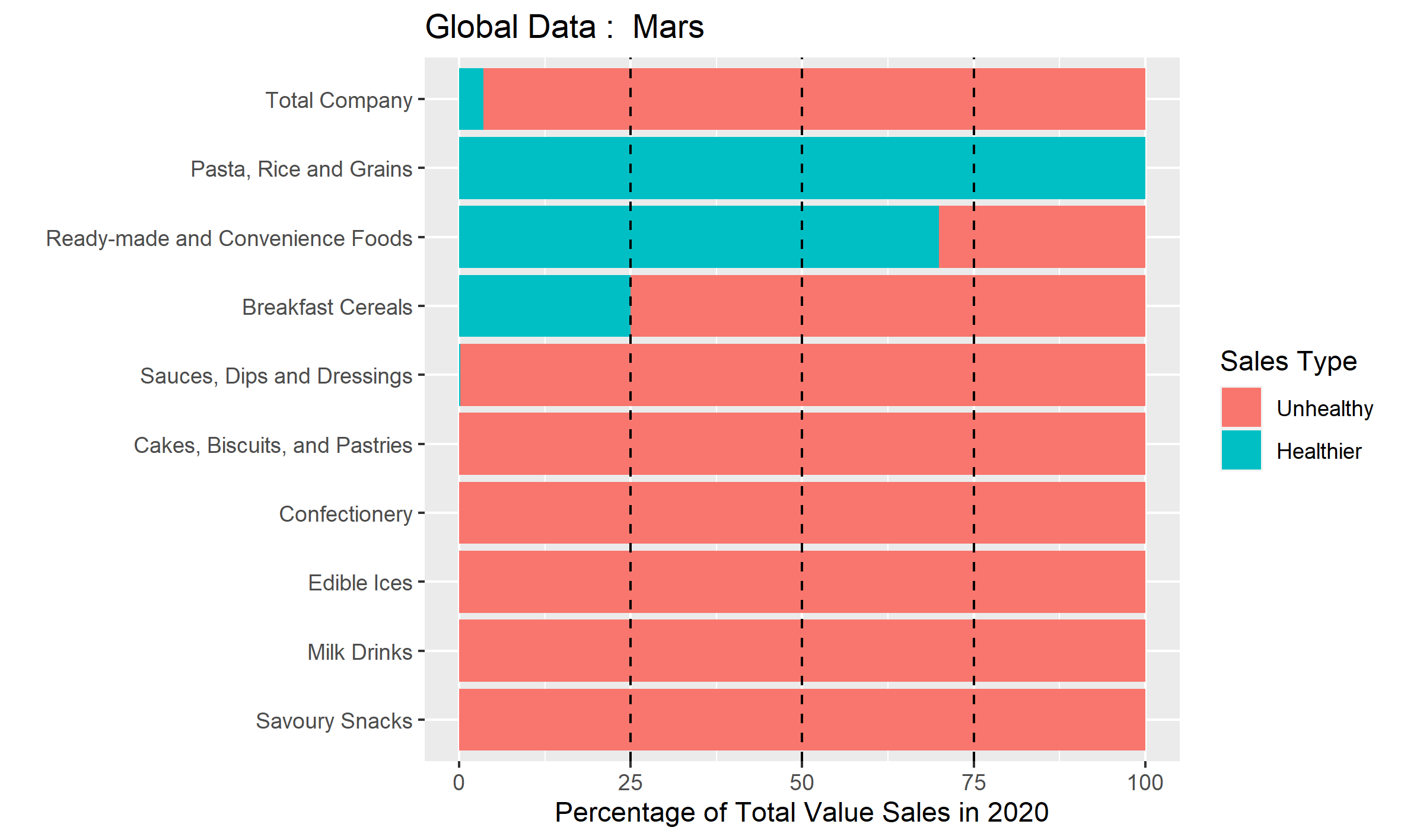
**

**
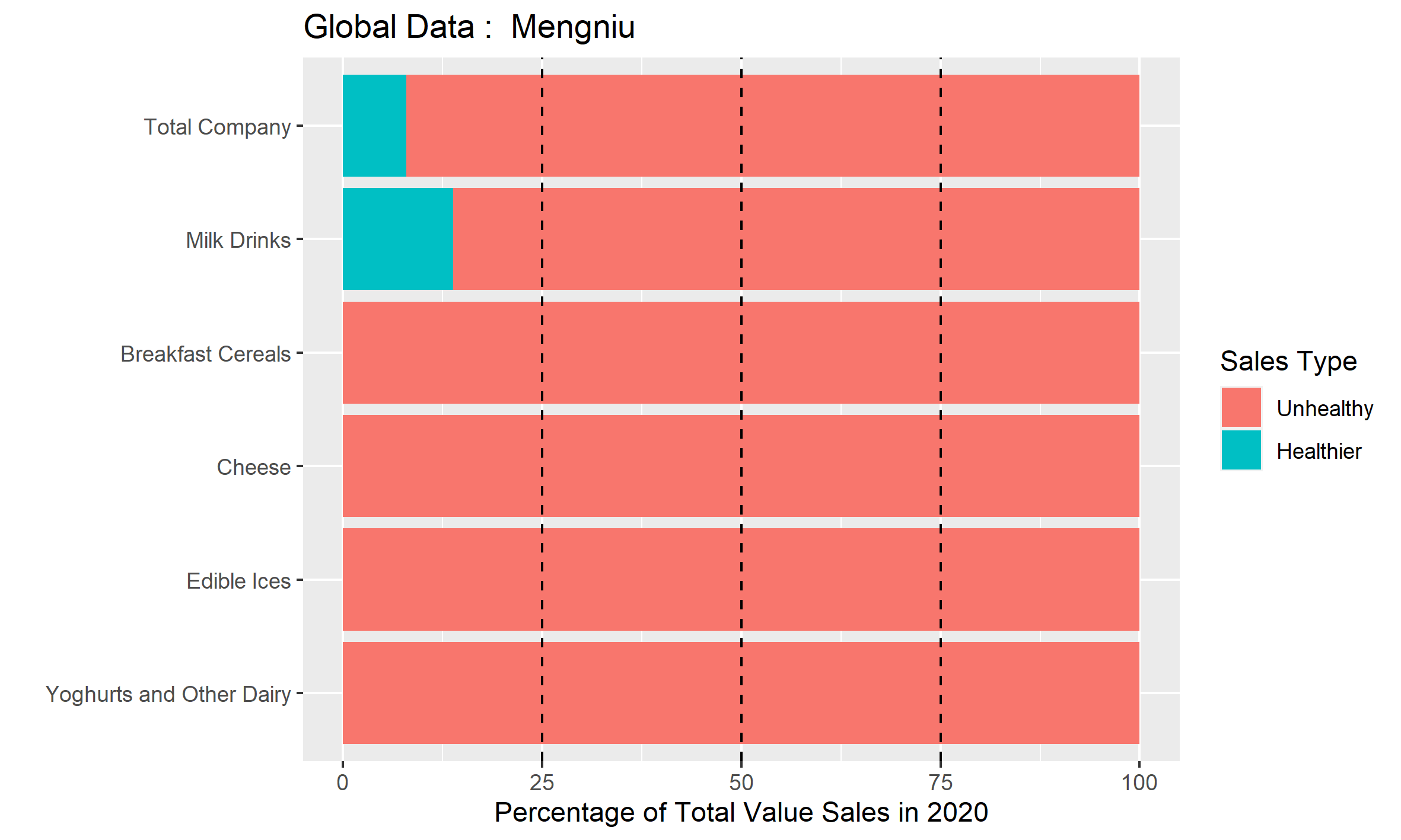
**

**
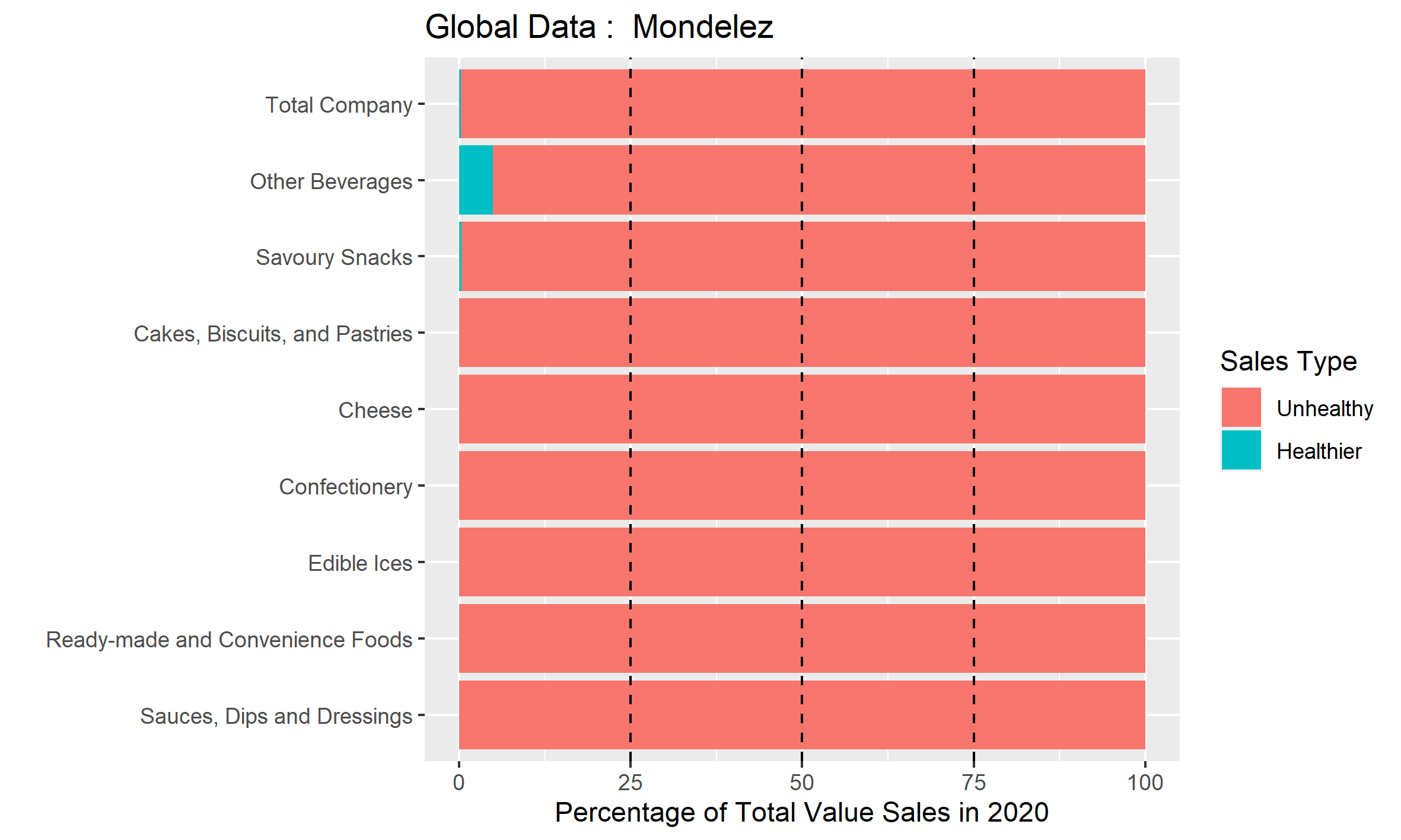
**

**
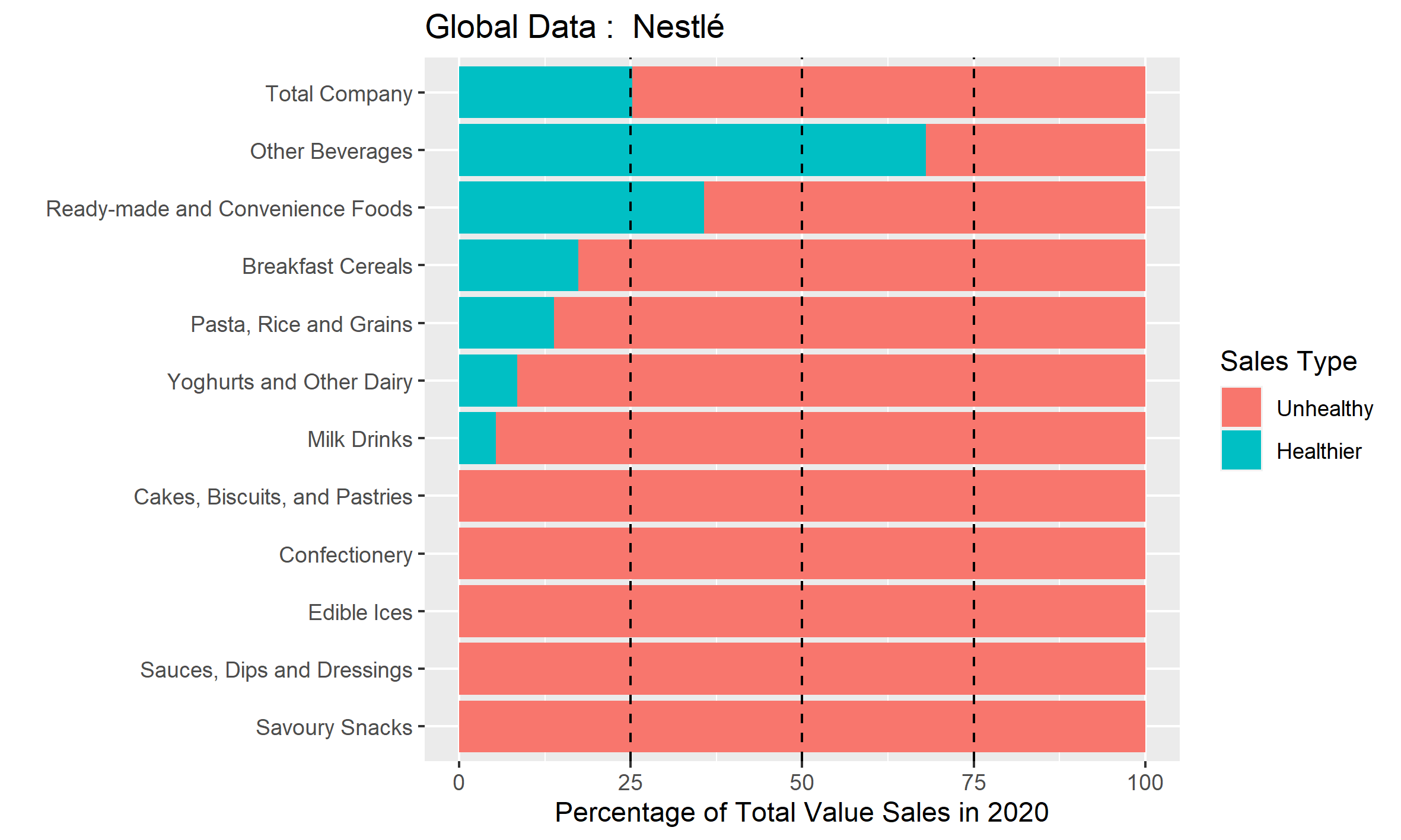
**


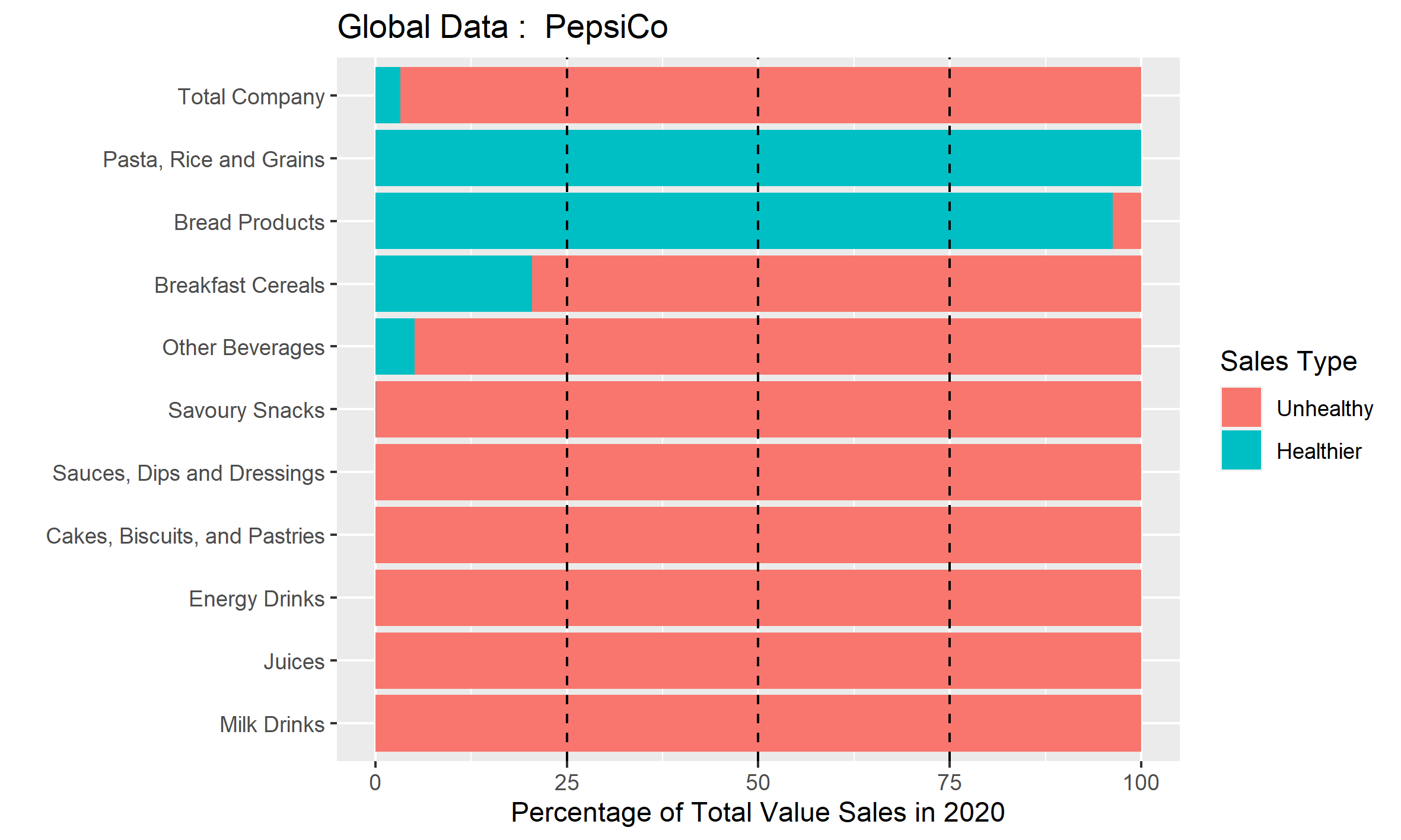


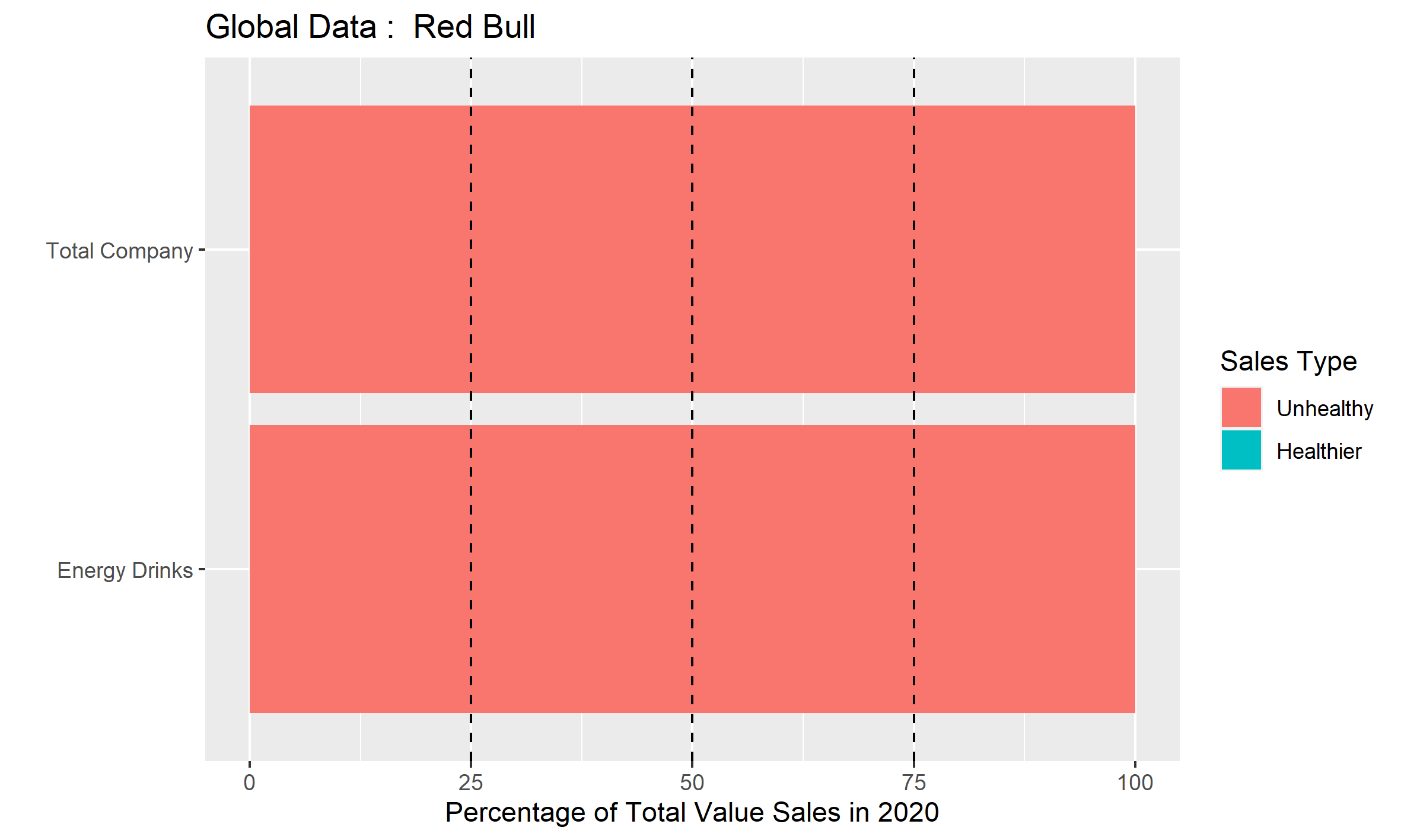


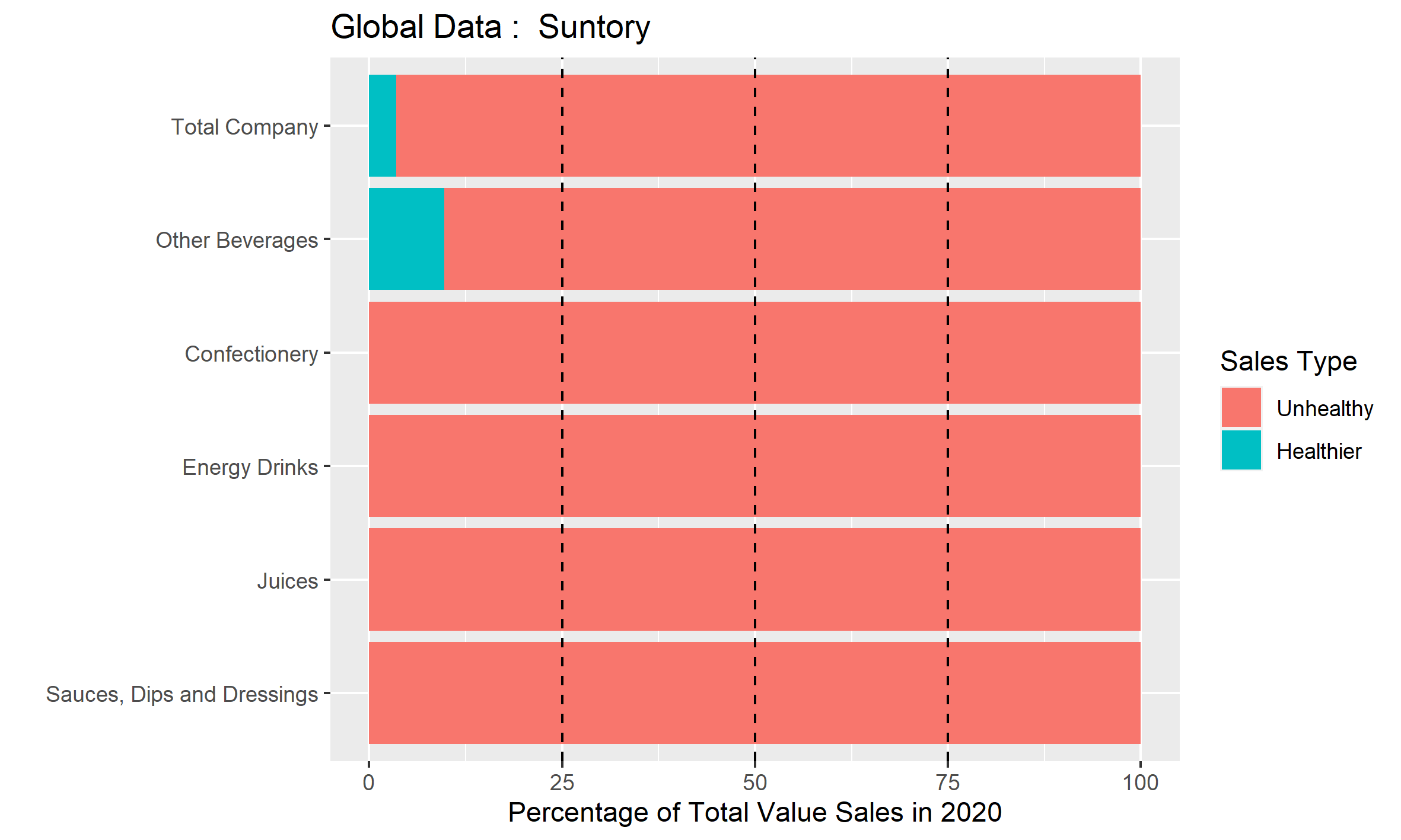


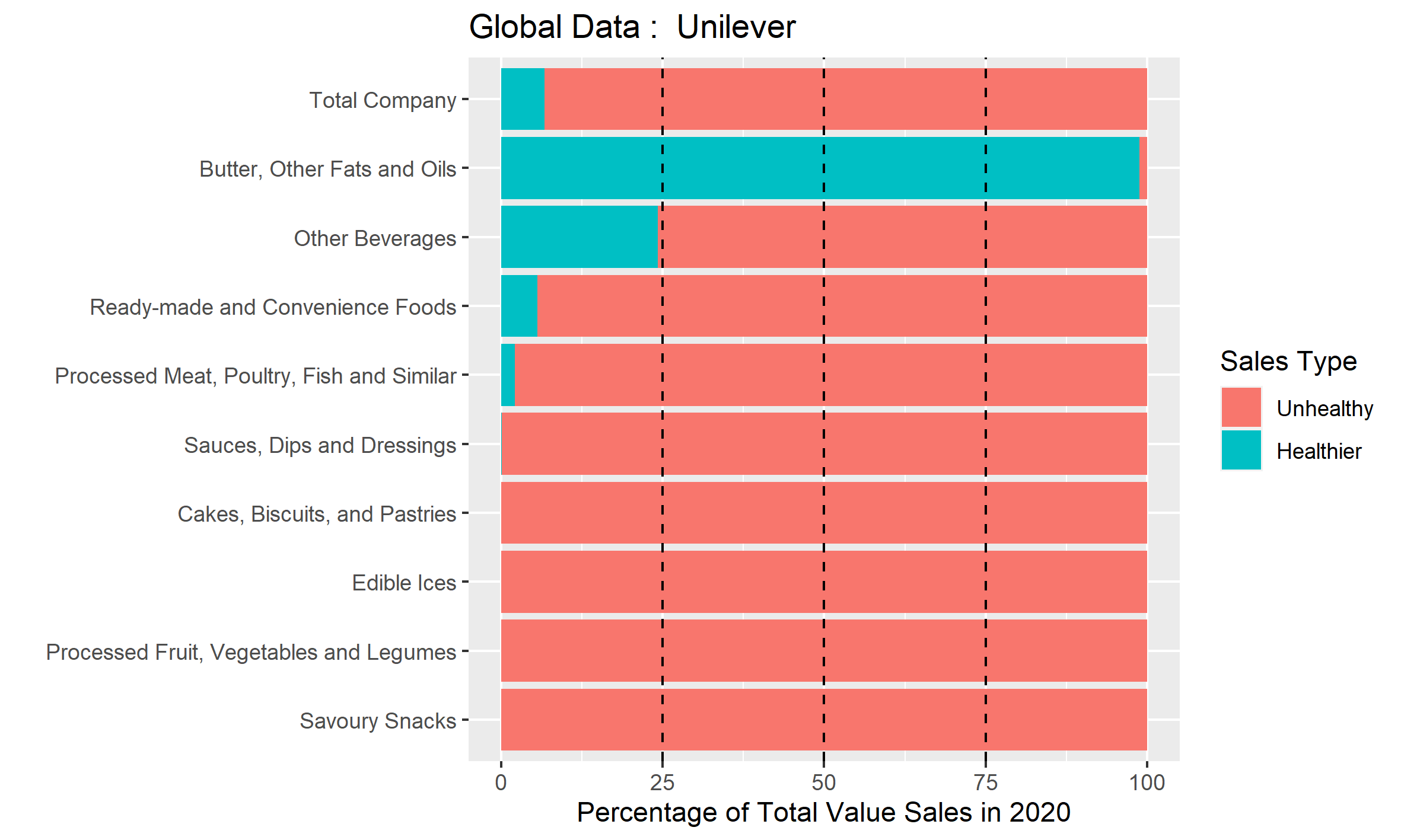


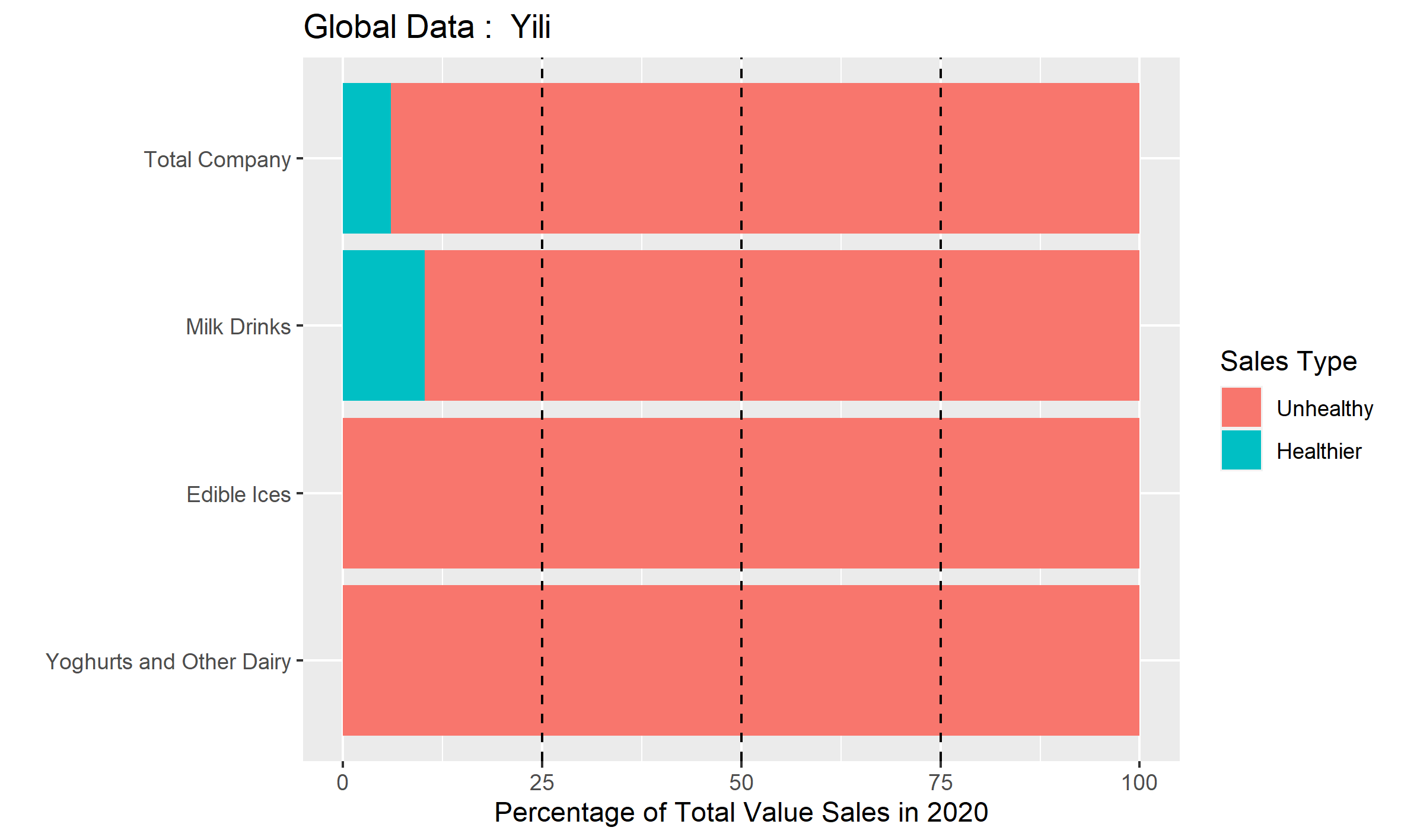

Supplement: Supplementary file 2 — Additional file 2. Proportion (%) of each company’s sales that are classified as unhealthy by category. [file 12992_2023_992_MOESM2_ESM.docx]
