# Supplementary material for: The development of a method for the global health community to assess the proportion of food and beverage companies’ sales that are derived from unhealthy foods
Source: Global Health. 2023 Dec 1;19:94. doi: 10.1186/s12992-023-00992-z (PMC10690999; doi:10.1186/s12992-023-00992-z)

## Additional File 3: Absolute sales classified as unhealthy by company and category

**Supplementary figure 1:** Absolute value sales (US$ million) that are unhealthy and healthier by company


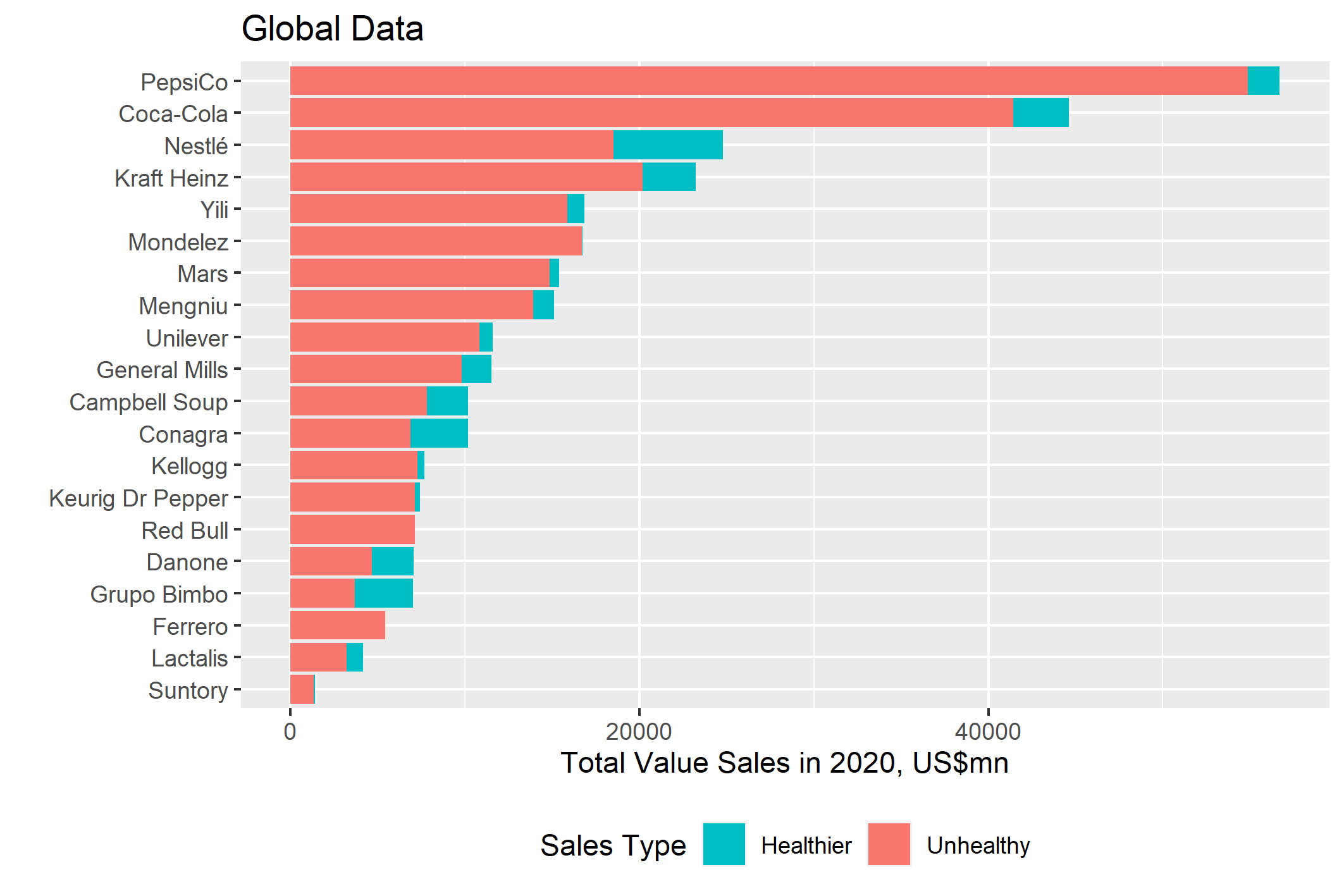


**Supplementary figure 2:** Absolute value sales (US$ million) that are unhealthy and healthier by category


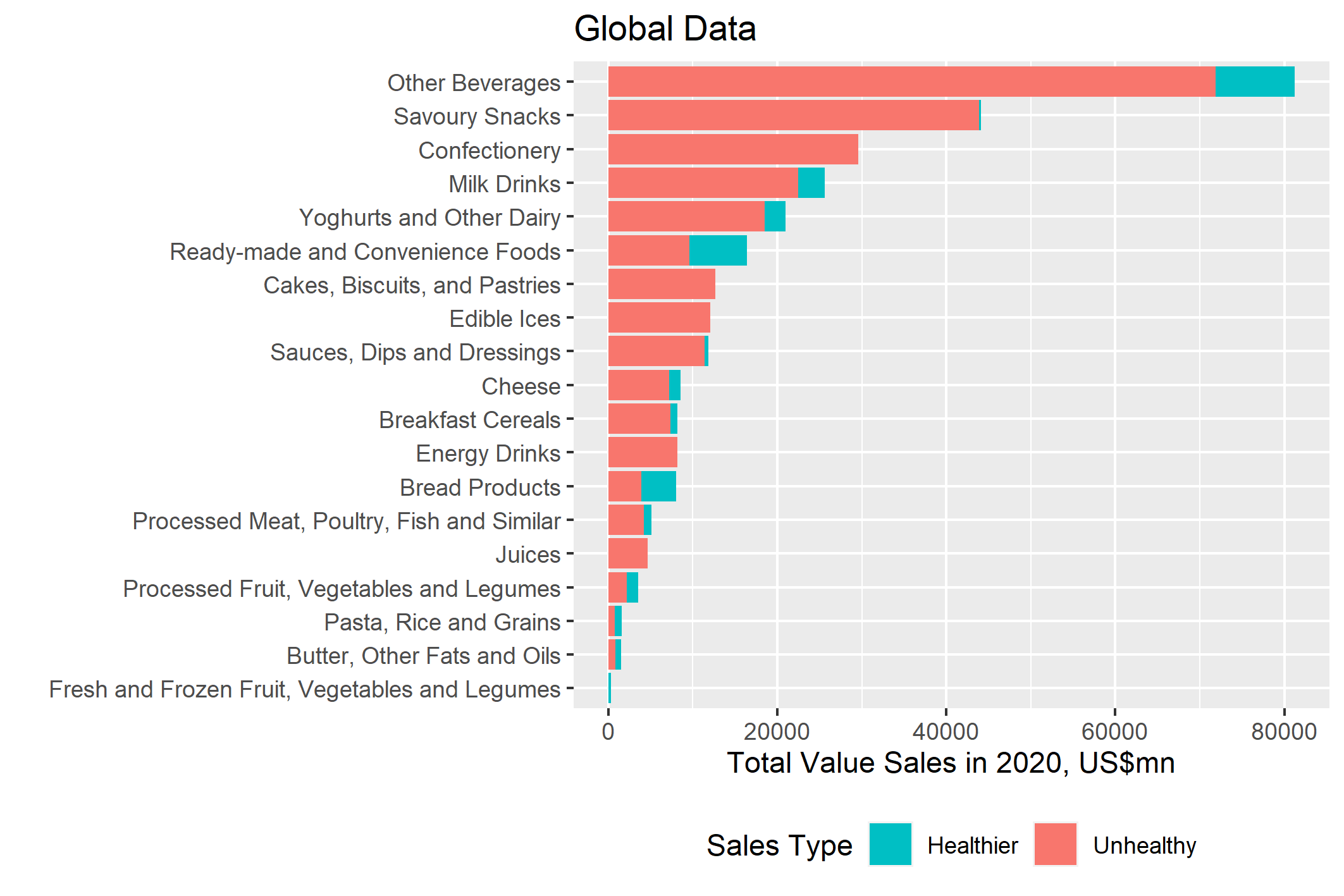

Supplement: Supplementary file 3 — Additional file 3: Supplementary Figure 1. Absolute value sales (US$ million) that are unhealthy and healthier by company. Supplementary Figure 2. Absolute value sales (US$ million) that are unhealthy and healthier by category. [file 12992_2023_992_MOESM3_ESM.docx]
